# A guide for studying among-individual behavioral variation from movement data in the wild

**DOI:** 10.1186/s40462-020-00216-8

**Published:** 2020-06-29

**Authors:** Anne G. Hertel, Petri T. Niemelä, Niels J. Dingemanse, Thomas Mueller

**Affiliations:** 1 Senckenberg Biodiversity and Climate Research Centre (SBiK-F), Senckenberganlage 25, 60325 Frankfurt am Main, Germany; 2 Department of Natural Sciences and Environmental Health, University of South-Eastern Norway, 3800 Bø i Telemark, Norway; 3 Behavioural Ecology, Department of Biology, Ludwig-Maximilians University of Munich, Planegg-Martinsried, Germany; 4 Department of Biological Sciences, Goethe University Frankfurt, Max-von-Laue-Straße 9, 60438 Frankfurt (Main), Germany

**Keywords:** Animal personality, Behavioral reaction norms, Behavioral specialization, Behavioral syndromes, Behavioral type, Biologging, Predictability

## Abstract

Animal tracking and biologging devices record large amounts of data on individual movement behaviors in natural environments. In these data, movement ecologists often view unexplained variation around the mean as “noise” when studying patterns at the population level. In the field of behavioral ecology, however, focus has shifted from population means to the biological underpinnings of variation around means. Specifically, behavioral ecologists use repeated measures of individual behavior to partition behavioral variability into intrinsic among-individual variation and reversible behavioral plasticity and to quantify: a) individual variation in behavioral types (i.e. different average behavioral expression), b) individual variation in behavioral plasticity (i.e. different responsiveness of individuals to environmental gradients), c) individual variation in behavioral predictability (i.e. different residual within-individual variability of behavior around the mean), and d) correlations among these components and correlations in suites of behaviors, called ‘behavioral syndromes’. We here suggest that partitioning behavioral variability in animal movements will further the integration of movement ecology with other fields of behavioral ecology. We provide a literature review illustrating that individual differences in movement behaviors are insightful for wildlife and conservation studies and give recommendations regarding the data required for addressing such questions. In the accompanying R tutorial we provide a guide to the statistical approaches quantifying the different aspects of among-individual variation. We use movement data from 35 African elephants and show that elephants differ in a) their average behavior for three common movement behaviors, b) the rate at which they adjusted movement over a temporal gradient, and c) their behavioral predictability (ranging from more to less predictable individuals). Finally, two of the three movement behaviors were correlated into a behavioral syndrome (d), with farther moving individuals having shorter mean residence times. Though not explicitly tested here, individual differences in movement and predictability can affect an individual’s risk to be hunted or poached and could therefore open new avenues for conservation biologists to assess population viability. We hope that this review, tutorial, and worked example will encourage movement ecologists to examine the biology of individual variation in animal movements hidden behind the population mean.

## Glossary

### Animal personality

Among-individual variation in average behavioral expression measured as the variance of a random intercept in a mixed -effects model. The existence and extent of among-individual variation is commonly quantified as repeatability (R).

### Behavioral type

An individual’s average behavioral expression, measured as an individual’s value of the random intercept of its reaction norm and respectively the individual’s position on the behavioral spectrum.

### Behavioral plasticity

Reversible changes in behavior in response to biotic and abiotic environmental conditions within the same individual.

### Behavioral syndrome

Correlation between an individual’s average expression of one behavior with its average expression of other behaviors in repeated measures data.

### Predictability

Among-individual differences in residual within-individual behavioral variability after controlling for variation in average behavior (behavioral type) and in individual plasticity.

### Reaction norm

Range of behavioral phenotypes that a single individual produces under different environmental conditions measured as the random intercept and slope of a random regression model. Behavioral plasticity exists when the reaction norm slope is non-zero. Individual variation in reversible behavioral plasticity (individual plasticity) exists when the reaction norm slope differs among individual.

## Introduction

Identifying the causes of individual variation in movement has been a key topic in movement ecology over the past decades [1]. Apart from variation due to life stage or sex, movement ecologists often examine individual variation in movement caused by external factors such as differences in the social and non-social environment, or variation in internal state such as hunger level or motivation to find a mating partner [2]. Animal behavior research suggests however that among-individual variation in behavior can not only be driven by external factors or internal state, i.e. reversible variation, but also by non-reversible intrinsic variation among individuals including differences in genetic make-up or developmental history (i.e. the social and non-social conditions experiences in the past), owing to research in a sub-field of behavioral ecology focusing on “animal personality” [3–5]. Such intrinsic individual differences in the mean expression of a behavior (i.e., animal personality) can have important ecological consequences, affecting predator-prey interactions [6, 7], population dynamics [8], dispersal [9] and survival [10]. Outside of movement ecology, animal personalities are studied in particular in the fields of ethology, animal behavior, behavioral ecology, and evolutionary ecology.

### Animal personality and the “two-step approach”

Animal personality is formally defined as repeatable individual differences in behavior across time and context [5, 11, 12]. In a seminal paper, Réale et al. [5] proposed a coherent terminology to study personality as five major behavioral traits – activity, exploration, boldness, aggressiveness and sociability. A huge body of literature has since used various experimental tests in the lab (e.g. open field tests) or built-in settings in the wild (e.g. flight initiation distance, novel object tests) to quantify individual variation in these behavioral traits [5, 13]. Experimental approaches have the big advantage of reducing bias of environmental variables which themselves create variation in behavior. In a second step individual variation in the experimental test is then linked to variation in natural behavior in the wild, including space use and movement behavior [14–20]. For example, “aggressiveness” in sleepy lizards (*Tiliqua rugosa*) affected space use, in particular under limited food availability [15]. This so called “two-step approach” is common even though it comes with some potential problems [21]. For one, to date there is little consensus how to unambiguously interpret behaviors measured using experimental tests [13, 22, 23]. More so, it has been questioned what inferences can be drawn from an artificial test situation when related to behavior in the wild [13, 21, 24]. Specifically, the “two-step approach” relies on the assumption that behavior expressed during the test situation correlates with behavior in the wild [21]. If the experiment is well designed to reflect the behavioral trait in question given the ecology of the species (reviewed in [13]), the insight gained from an experiment can serve as a valuable baseline to relate space-use in the natural environment to [15]. However, the behavior in a potentially stressful artificial context, such as during capture, handling or testing in a novel environment, may not always reflect or appropriately correlate with behavior in the wild [25, 26]. Finally and importantly, experimental approaches may be logistically or ethically impractical to apply with larger, elusive, or endangered wildlife [18, 27].

### Using variance partitioning to study individual variation in movement

An alternative approach to the study of animal personality, particularly pertinent in behavioral ecology and evolutionary biology, is that over repeated measures taken in different biological contexts and over some portion of an animal’s lifetime, individuals can differ in their average expression of *any* kind of behavior [28, 29]. This approach is purely based on statistical partitioning of behavioral variation into its environmental, among-, and within-individual sources [30] and is neither semantically nor methodologically constrained to the five major behavioral traits. By “focusing on observable patterns *per se*” [28] this approach can distinguish intrinsic individual variation from reversible behavioral plasticity but avoids confusion over the proximate causes of individual variation (e.g. genetics, developmental, social). As an addition to this approach, personality (i.e. among-individual variation) in multiple observable behaviors can be underpinned by a common latent (i.e. unobservable) variable such as aggressiveness or boldness [31]. However such latent variables can only be identified through appropriate statistical estimation of a correlation matrix at the among-individual level, which is then taken forward in Structural Equation Models (SEM, [31, 32]). This is, however, beyond the scope of our present work. This paper adopts the definition of animal personality as statistical partitioning of behavioral variation, and focuses on providing a framework where individual variation in space-use and activity behaviors can be studied directly from movement data. Biologging, accelerometer, and tracking data used in movement ecology are exceptionally suitable to study variation in behavior at the individual level: automated tracking devices produce large, continuous datasets of individual-based measurements which code for behaviors like habitat use, movement, (diel) activity, or detailed behaviors of animals over meaningful time scales [33–35].

### Aim and scope

The first aim of this paper is to provide a literature review of studies that used variance partitioning to study different aspects of individual variation in movement behaviors. Indeed, variance partitioning has already been used to study individual variation in foraging behavior of marine mammals and birds [36–38], in fish activity and movements [39–42], in movement and habitat selection of terrestrial mammals [27, 43–46], and in partial migration strategies of bats, birds, fish, and mammals [47–50]. Despite this growing body of literature, research concentrating on individual variation from movement data has developed quite isolated within the different animal taxa and with this systematic review we hope to facilitate a synthesis of existing efforts.

Our second aim is to introduce the concepts, terminology, and statistical approaches used in behavioral ecology to describe individual variation at different hierarchical levels. Last, we aim to demonstrate in a hands-on R tutorial [51] and with example data, how movement ecologists can use statistical tools adopted by behavioral ecologists to partition variation in movement data into environmental, among-individual, and within-individual sources. We believe that this approach is widely applicable and could open new avenues to study biological variation in movement across all kinds of movement forms and animal taxa.

## Evidence for among-individual variation in movement behaviors in the wild

Movement ecologists often seek to find patterns in animal behavior owing to differences among individuals in e.g. life stage, sex, age, the social or non-social environment or reversible internal states (reviewed in 1). Yet, among-individual differences in movement behavior beyond such effects are common [1, 52]. We here review the evidence for among-individual variation in movement patterns within populations spanning movement of avian, aquatic, and terrestrial taxa.

If individuals differ in their movement and space use behavior over a significant portion of their lifetime, then the behavioral niche occupied by single individuals is much smaller than the population niche: a case of behavioral specialization [53]. Most examples of movement behavior specialization study among-individual variation and within-individual consistency of foraging strategies in marine mammals and birds (e.g. [36, 37, 54, 55]). In central place foragers, where individuals return to a fixed location between foraging trips [56], each foraging trip can be described using variables such as trip duration, distance, duration of diving, departure angle, and the longitude and latitude of the endpoint of the trip [36–38]. Single individuals thereby show remarkable specialization in how and where they forage across repeated trips but differ in their foraging strategy from each other [36–38]. Additionally, some populations harbor a mix of foraging specialists and foraging generalists [38, 57, 58]. Foraging site fidelity is a metric that has been used to capture an individual’s degree of foraging specialization, where individuals either use always the same site for foraging or are variable in where they forage [38, 59]. Indeed, differences in the degree of foraging site fidelity have been linked to age or sex differences [60, 61], differences in flight response to human encounters [62], and may have consequences for individual foraging efficiency and mass gain under varying climate conditions [54]. In marine and pelagic fish, advances in acoustic telemetry systems have led to a number of studies demonstrating repeatable individual variation in e.g. home range size, daily movement distance, diel vertical migration, site fidelity, and dispersal of fish [39–42]. A few recent studies in terrestrial mammals show that individuals within populations differ in movement patterns, diel activity, and habitat selection preferences [27, 44–46, 63] and in their behavioral plasticity along resource gradients [46]. Differences in behavior in relation to risk-benefit trade-offs and the thereof resulting consequences for individual fecundity and survival have received particular attention. For example, for ungulates, more open habitats increase the risk of detection by predators, including human hunters, but may at the same time provide good foraging opportunities, a trade-off that some individuals are more likely to accept than others [43, 64, 65]. As a consequence these individuals may experience higher fecundity [17] but also higher mortality because their behavior increases their predation likelihood [64, 66]. Increases in predation pressure, for example when hunting quotas are increased, may disproportionally increase the removal of individuals displaying risk-enhancing behaviors [67]. If individuals distinctly differ in their habitat selection, movement, and activity patterns this leads to spatial structuring of populations [68]. Such spatial structuring has even been observed in species which traditionally have synchronized long distance migrations. In elk (*Cervus canadensis*), socially dominant and habituated individuals have been shown to switch their migratory strategy and become resident in the vicinity of urban areas, as opposed to their less habituated conspecifics [50]. This demonstrated that individuals with movement and habitat selection strategies contingent with habituation may adapt better to anthropogenically altered landscapes [69].

The non-random distribution and local clustering of behavioral types in space has numerous other important consequences for ecology and evolution (see [70] for an extended review) which could be explicitly studied using movement data in the wild. For example differential habitat specialization may lead to habitat specific differential susceptibility to encounter conspecifics, disease vectors, or prey [70, 71]. Ultimately, individual variation and specialization in behavior, habitat, and resource use are expected to decrease competition and increase population productivity and carrying capacity [72].

## Key concepts in behavioral ecology research focusing on individual differences

Behavioral ecologists focusing on individual differences in behavioral expression seek to quantify sources of variation in behaviors and to study the ecological and evolutionary causes and consequences of such variation [3, 4, 70, 73–75]. There are three principal ways in which behavior can vary among individuals. Individual variation in average behavior over repeated observations (“personality”), and individual variation in degree of behavioral plasticity towards changing environmental conditions (“individual plasticity”) can be jointly quantified by adopting a reaction norm framework [29, 76]. A reaction norm is formally defined as the range of behavioral phenotypes that a single individual produces along a given environmental gradient [29]. The individual’s “behavioral type” is its intercept of the reaction norm and the slope is its individual “plastic” response to changes in the environment. Thirdly, there can be individual variation in residual within-individual variance (“behavioral predictability”), i.e. the “deviation” from this reaction norm [77]. Finally, among-individual correlations between all three variance components can exist. For example, behavioral syndromes represent between-individual correlations of two or more distinct behaviors in repeated measures data [78, 79].

### Behavioral type

**Fig. 1:**
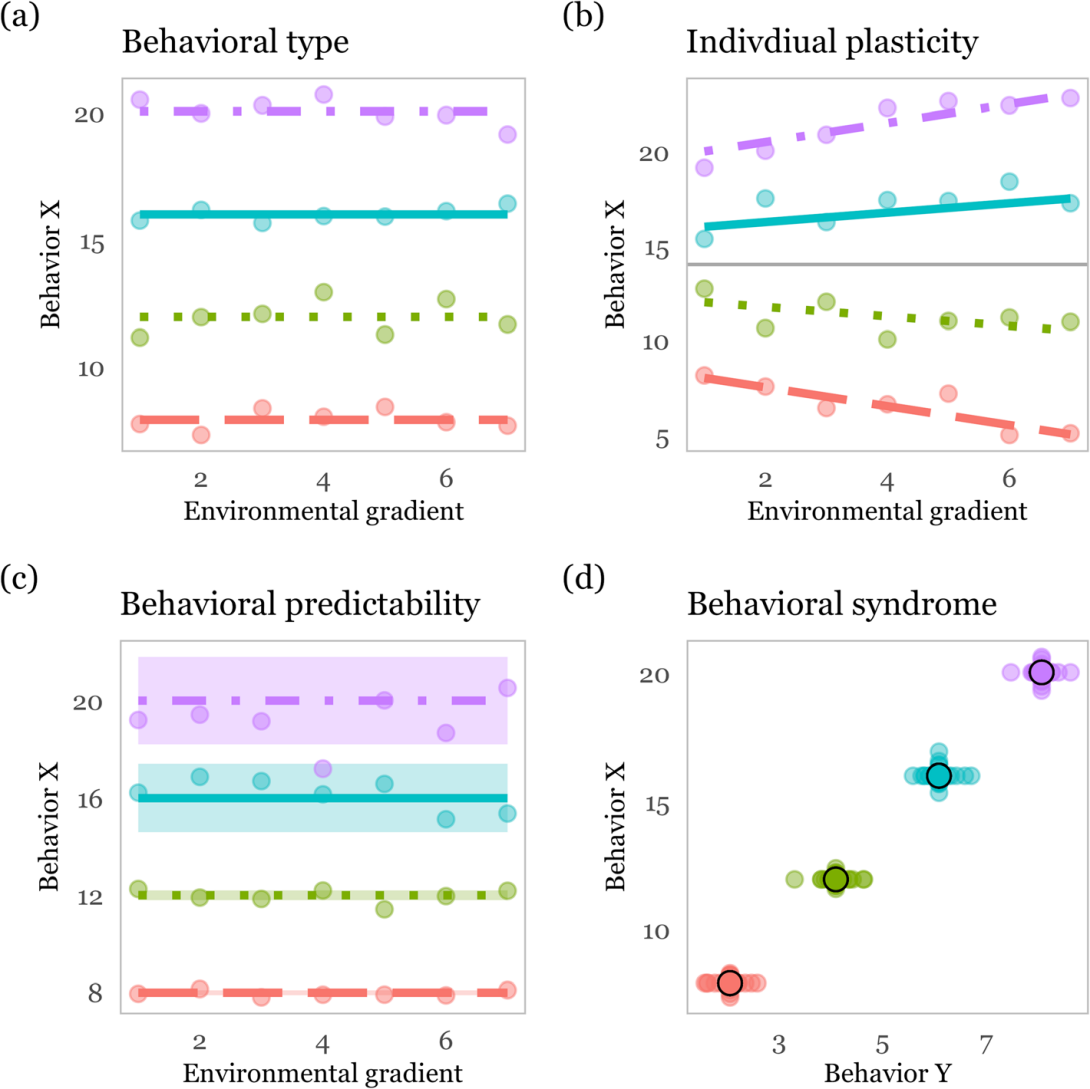
Concepts in animal personality research: **a** Behavioral types: Among-individual differences in mean behavioral expression over repeated measures. **b** Linear reaction norm plot: individuals differ in their behavioral plasticity (slope) along an environmental gradient and there is a positive correlation between an individual’s behavioral type (intercept) and its plasticity (slope). **c** Predictability: individuals differ in within-individual behavioral variability from more predictable individuals (red ribbon) to less predictable individuals (purple ribbon). **d** Behavioral syndrome: there is a positive among-individual correlation for two distinct behaviors: Behavior X and Behavior Y. Individuals with on average higher average expressions of Behavior X also have higher average expressions of Behavior Y

When individuals differ over repeated measures of a behavior from each other, then each individual only expresses (at least most of the time) a limited range of the behavioral expression present in the population and varies in its average behavioral expression, i.e. its “behavioral type”, from other individuals in the population (Fig. 1a, 5). The extent of individual variation in a population is commonly quantified as ‘repeatability’ (R), where among-individual variance is standardized by the total phenotypic variance, ranging from 0 to 1. Repeatability indicates the proportion of phenotypic behavioral variance in a population that can be attributed to individual differences in behavioral expression [80]. Across animal taxa and behaviors (both in the laboratory and the wild), differences between individuals account on average for approximately 40% of the total behavioral variance [80, 81]. However, repeatability cannot generally be compared across groups of animals to make interpretations about whether those groups differ in the expression of among - individual variation because repeatability also varies as a function of the within-individual variation [82]. Instead the coefficient of variation for among-individual variance (i.e. CV_i_), allows for comparisons of degree of among-individual variation across populations (or any other groups of animals) [81, 83]. CV_i_ is calculated as the among-individual variance standardized by the trait mean [81]. Both repeatability and CV_i_ are population-level parameter estimates of the degree of individual variation. On the individual level, an individual’s ‘behavioral type’ (the random intercept of the reaction norm) indicates its relative position on the behavioral spectrum of the population (Fig. 1a) and can be visualized by extracting the individual’s best linear unbiased predictor (BLUP) or posterior distribution from a mixed effects model [84]. Using movement data, among-individual differences in behavioral types have been demonstrated for example for swimming activity, diel vertical migration, horizontal movement, activity level, home range size, site fidelity, and dispersal of fish [39–41]. In the terrestrial literature, a few studies have demonstrated that individual variation and hence specialization in habitat selection can be significant and mask selection patterns on the population level [44, 63, 85, 86]. Movement data can also be used to quantify diel activity patterns and recent studies have demonstrated significant individual variation in diel activity strategies within populations suggesting intra-specific temporal niche partitioning [87, 88].

### Individual behavioral plasticity

In addition to the ‘behavioral type’, behavior may also be context-dependent and change with life history stage, environmental conditions, or over time. In behavioral ecology this type of plasticity is referred to as reversible ‘behavioral plasticity’. How animals adjust movement along environmental gradients or over time is of general interest in movement ecology but individual variation in the degree of adjustment (i.e. individual plasticity) is rarely evaluated explicitly (but see [46]). Limited behavioral plasticity may thereby be indicative of behavioral specialization. A few studies have demonstrated the existence of individual variation in reversible behavioral plasticity (the random slope of the reaction norm) in response to environmental gradients (Fig. 1b), in movement behaviors [40, 46]. Perch, for example, increased swimming activity in response to increasing water temperature but individuals differed in their degree of behavioral plasticity leading to increased individual differences in swimming activity at higher temperatures [40]. Individual variation in plasticity is best studied using random regression models on repeated behavioral measures across environmental conditions or time [30, 89]. These models allow the simultaneous estimation of 1) the individual variation in the average behavioral expression (i.e. behavioral type; intercept of the reaction norm) and 2) the individual variation in the change of behavior over environmental gradients (i.e. individual plasticity, reaction norm slopes).

### Behavioral predictability

In addition to variation in behavioral plasticity, individuals may differ in their degree of residual within-individual behavioral variability [90], also termed predictability [91]. This kind of variation represents the unexplained variance of repeated behavioral measures after controlling for individual variation in average behavior and in individual plasticity (Fig. 1c). Unpredictable individuals are characterized by high variability around their average behavioral type and reaction norm slope. Predictable individuals on the other hand have little residual variance around their behavioral type and reaction norm slope. Among-individual variation in predictability can statistically arise due to incomplete or erroneous model specification where we fail to account for plastic responses towards environmental covariates [77]. However, individual variation in predictability may also be evolutionarily adaptive, e.g. diversification in bed-hedging [92] or food intake rates [93]. Certain conditions may therefore favor the coexistence of more and less predictable individuals (see [77] for an extended review). When important social- and nonsocial environmental covariates are controlled for, predictability can be interpreted as a measure of an individual’s degree of behavioral specialization relative to other individuals with predictable individuals being more specialized on a certain behavioral expression. Both, behavioral plasticity and behavioral predictability can thereby constitute different drivers of behavioral specialization. Some movement behaviors, the degree of foraging site fidelity for example, could be considered as inherently coding for intra-population variation in within-individual variability [54, 58, 62]. In these examples, more site-faithful individuals feature low within-individual variability in space use, i.e. they repeatedly visit the same locations for foraging, whereas less site-faithful individuals have high within-individual variability in space use and can switch foraging sites [58]. Alternatively, recent advancements of classical mixed effects models to double-hierarchical generalized linear models (DHGLM’s) allow for inferences about statistical predictability in any kind of behavior, by simultaneously estimating both the variation in the individual mean and the variation in the residual variance around the individual mean within the same model [94]. For example, variation in predictability has been shown for activity rates in guppies (*Poecilia reticulate* [95]) and exploration of open habitat in sticklebacks (*Gasterosteus aculeatus* [96]) but has not been evaluated in movement data from the wild.

### Correlations among variance components and behavioral syndromes

Correlations between all three variance components described above are possible. Correlations between behavioral type and individual plasticity (i.e. intercept-slope correlation, [29, 97, 98]) for example can give insights whether individuals of a higher average behavioral expression adjust their behavior more (or less) strongly as compared to individuals with a lower average behavioral expression and whether among-individual variance and repeatability change over the environmental gradient (Fig. 1b). Caribou (*Rangifer tarandus*) for example show behavioral type-dependent plasticity, where fast moving individuals in an average environment decreased movement speed more strongly with increasing resource aggregation than individuals that moved more slowly [46]. In the same manner, both the behavioral type and the behavioral plasticity can also be correlated with behavioral predictability, where individuals at one end of the behavioral spectrum and/or ones with limited (increased) behavioral plasticity are also more or less predictable [96]. To our knowledge such correlations have so far only been shown for movement traits measured under laboratory conditions [96]. Finally, the individual’s average expression of one behavior can be correlated with the average expression of other behaviors, termed ‘behavioral syndrome’ (4, Fig. 1d). Traditionally, behavioral syndromes are defined as the correlation of behaviors at the individual level measured in different contexts, for example the behavior in a novel object and the behavior in an open field test [4]. In empirical, non-experimental, movement data the context is often not defined and behavioral syndromes estimated from these data possibly present the average behavior over a number of contexts (foraging, routine movements, predator escape) rather than from different contexts. Movement behaviors of wild burbot (*Lota lota*) for example correlated in a behavioral syndrome ranging from resident individuals with small home ranges, low movement activity and high site fidelity, to mobile individuals with large home ranges, much movement and low site fidelity [41]. Because there is strong support that movement behaviors are heritable [99, 100], with increasing evidence for heritability of natural movements in the wild [101, 102], correlations of distinct movement behaviors thus also have a genetic basis [103]. Correlated behaviors or traits can enhance or restrict a population’s capacity to adapt to changing conditions [70, 104]. Among-individual correlations are not restricted to behavior but can also include correlations with morphology [105] or with life history traits, termed ‘pace of life syndrome’ (POLS, [75, 106]).

## How to disentangle intrinsic individual variation from reversible behavioral plasticity

**Fig. 2:**
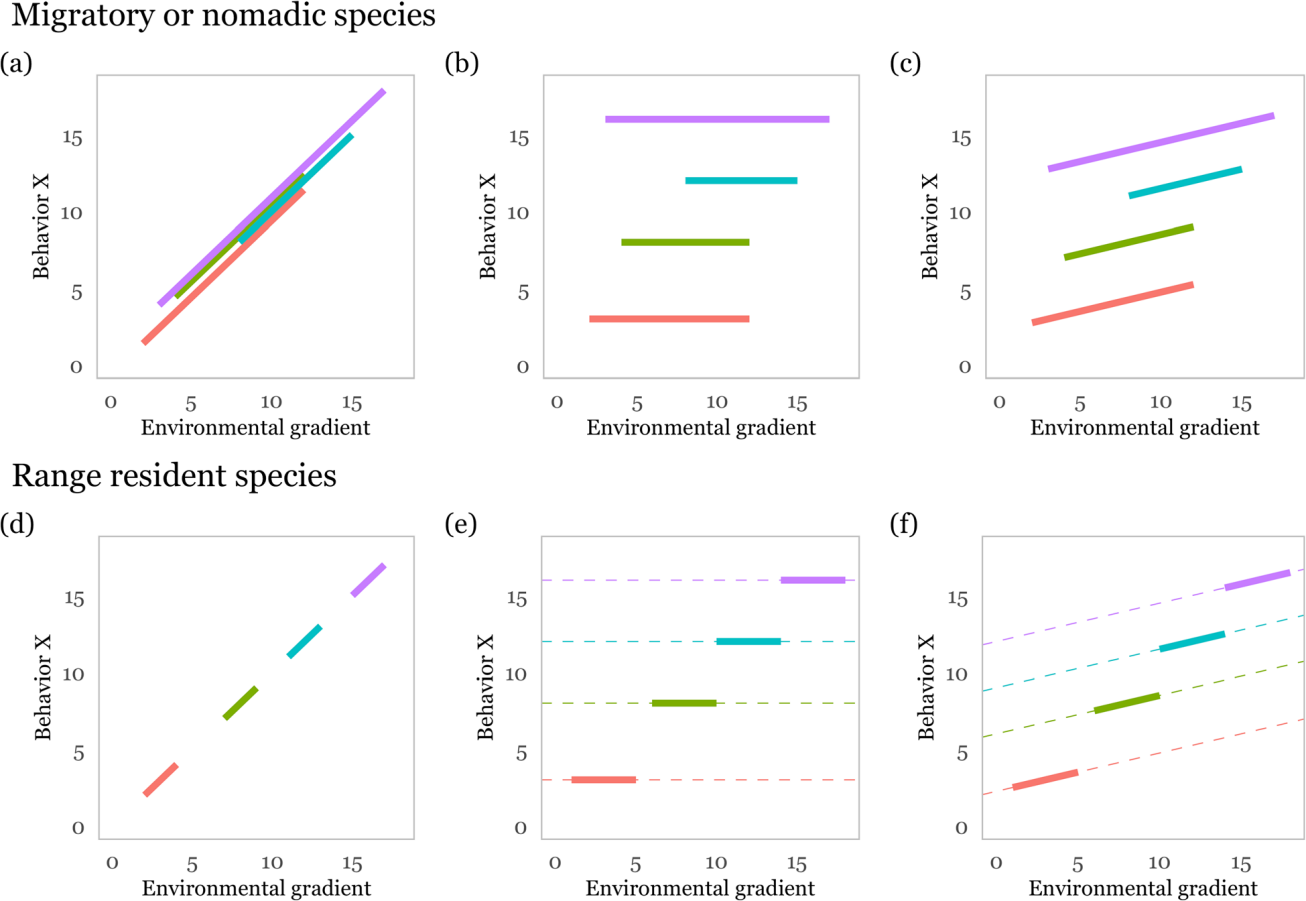
Possible relationships between behavioral types (among-individual variation in intercepts) and behavioral plasticity (non-zero slopes) along environmental gradients for mobile (**a**, **b**, **c**) and stationary (**d**, **e**, **f**) species. Mobile species may be exposed to a wider gradient of environmental conditions than range resident species, allowing to disentangle behavioral plasticity and behavioral types more easily. Individuals may adjust their behavior plastically to the local environment while not differing in behavioral type (**a** & **d**). Behavioral differences exist when individuals are not observed over the same environmental gradient (**d**). Alternatively, individual differences may fully account for behavioral differences with no behavioral plasticity to environmental conditions (**b** & **e**). In stationary species this may lead to non-random distribution of behavioral types when individuals choose habitats which match their behavior (**e**). Most likely, individual differences and behavioral plasticity to environmental conditions jointly contribute to observed behavioral differences (**c** & **f**). This figure has been adapted from Sprau and Dingemanse [107] and Niemelä and Dingemanse [26]

An inherent problem in studies that record behavior in the wild is that individuals always experience at least subtly different environments precluding the option to measure them under identical conditions [52]. This poses difficulties for attributing observed behavioral differences to differences in genes and developmental history (i.e. intrinsic variation) or to prevailing environmental conditions. If the differences in the environment under which the animals’ behavior is assayed cause individual differences in behavior (via reversible behavioral plasticity towards the environment) and we fail to control for this, we might conflate individual and environmental variation (Fig. 2a&d, [26]). The key to account for the effect of environmental heterogeneity on individual variation is to repeatedly measure behavior of the same individuals over a range of environmental conditions. Ideally one can measure the behavior of individuals along similar environmental gradients to disentangle true from pseudo-repeatability (Fig. 2b&c, [26, 108]). Essaying movement behavior along environmental gradients such as habitat type or human disturbance may be easier for nomadic or migratory animals that move along such gradients (Fig. 2a-c), although this may not be true for covariates driving migration, such as climate or vegetation phenology [109]. For animals that maintain stable home ranges, environmental gradients may occur within their home range or manifest over time (Fig. 2d-f). However, in range resident species, individuals may match settlement choices to their behavioral type and hence only occur in certain environments (Fig. 2e&f, [9, 16, 110]). A way to disentangle intrinsic from environmentally induced behavioral variation is to include repeated measures of the environmental conditions (e.g. habitat availability, composition, structure, or temperature) at the time and location where behavior is measured or identity of the environment (e.g. territory), as fixed [107] or random [26] effects in mixed-effects models, respectively. One can then statistically distinguish plastic responses to the environmental gradient from patterns of non-random distributions of behavioral types (Fig. 2c) even if individuals have not been measured over the full environmental gradient (Fig. 2f, [107, 111–113]). For example, individuals may adjust their behavior in response to changing local conditions but beyond that still differ markedly in their behavioral type (Fig. 2c&f).

While the techniques above may account for important environmental variability, we usually do not possess information about all aspects of the social or non-social environment. This is especially the case for high-resolution tracking data where environmental variables are rarely recorded at the same spatial and temporal scale as the movement behavior. To help gauge whether it is likely that some important covariates have been left out, for example when spatial heterogeneity in the environment is expected but not explicitly mapped, one can test whether behavioral tactics are spatially autocorrelated, i.e. whether behavioral measures taken close in space are more similar than behavioral measures taken at a distance [66, 86]. Spatial segregation of behavioral tactics may thereby be informative in its own right [86]. On a similar note, movement data are inherently temporally autocorrelated, meaning that the behavior of an individual on a given day is likely to be more similar to its behavior on the previous day than to its behavior three weeks ago. Ignoring temporal autocorrelation can bias estimates of individual variation upwards even after controlling for temporal trends [114]. Using auto-regression structure one can calculate the correlation between residuals of behavioral measures taken at successive points in time (e.g. days, [115]). Temporal autocorrelation can be avoided when repeated behavioral measures are taken spaced out in time, and including a sequential effect of time (e.g. month, season) in the model should sufficiently control for temporal effects. Testing for different temporal scales of repeatability, i.e. long-term and short-term scale, can elucidate whether individual differences are short lived and hence potentially driven by the environment or long lived and hence more likely to be intrinsic to the individual [98]. Additionally to the non-social environment, demographic or social dependencies are another example how experienced environments may differ among individuals and may generate individual differences in behavior [116]. Social interactions may either promote [117] or dampen [118] behavioral variation, in either way individuals belonging to the same social group may be non-independent from other members of the group [119]. When social groups are dynamic, for example in fission-fusion societies, measuring behavior repeatedly over long time scales may facilitate teasing apart behavioral adjustments towards conspecifics and individual behavioral consistency [120]. Even solitary animals may still adjust their behavior in relation to local conspecific density (or to their respective predator or prey density) to facilitate temporal or spatial niche partitioning [19, 87]. Demographic traits such as sex, age, and reproductive status, or life history stage may all drive individual differences in behavioral expression and should therefore be controlled for [60]. In some cases, one may concentrate on a homogenous group of individuals (e.g. only adult females) to exclude known sources of behavioral variability or to study the extent of variation within specific age or life history classes [121]. Further, individuals may differ in a whole variety of other stable or labile “internal states” including for example hunger level, parasite load, or energy reserves [122] which can affect their motivation or capability to move [2]. It is virtually impossible to control for all internal and external aspects affecting behavior, even under laboratory conditions [123]. Taking repeated behavioral measures across multiple ecologically important contexts, across environmental conditions, and over long time periods facilitates disentangling individual effects from behavioral plasticity and reduces the effect of environmental noise [26, 124]. Tracking data thereby offer unique opportunities to obtain such long-term monitoring of behavior with virtually hundreds of repeated behavioral measures per individual.

Ultimately, the relative contribution of genetic versus permanent environmental effects on among-individual variation can only be assessed by quantifying heritability of movement behaviors [99, 101, 125]. We here gave recommendations how empiricists can disentangle individual variation from reversible behavioral plasticity [26]. We want to stress that results from studies that cannot control for all important co-variates that cause reversible behavioral plasticity are still meaningful as long as possible limitations on study conclusions are appropriately addressed.

## Data requirements to measure individual variation in movement behavior

While data requirements are always question and system specific, we here provide a few examples and general recommendations on the type of data needed to estimate individual variation in movement using variance partitioning.

Three prerequisites and challenges to the study of individual behavioral differences from movement data are that:

1. The temporal resolution of the movement data needs to be adequate to detect the focal behavior and comparable across all individuals. Many behaviors are short term and thus require high temporal resolution (short GPS fix interval) to be measured with movement data, e.g. area restricted foraging [36].
2. The behavioral measures need to happen with sufficient amount of repeats [82] but repeated measures can stretch out over a long time frame relative to the life span of the individual and hence require long monitoring durations. Ideally behaviors are observed across a significant portion of an individual’s lifetime or are spaced apart sufficiently to avoid pseudoreplication.
3. A sufficient sample of individuals from the same population are measured. The more individuals are followed the easier it is to statistically estimate individual level patterns.

While data with a high resolution and long duration over many individuals in a population become increasingly available, this traditionally tends to present a major financial and ethical challenge for movement ecologists. However, not in all cases all conditions (long duration, high resolution, many individuals) need to be met. For example, questions related to annual breeding dispersal distance and breeding site fidelity, or migration patterns require multiannual time series allowing to model individual level variation in behavior over long time spans (Table 1). Estimating individual differences of such broad scale movement patterns on the other hand only requires long GPS fix intervals at the scale of days or even months. At the other extreme we may need short GPS fix intervals to quantify individual variation in the degree of area restricted search behavior (ARS), the duration an animal keeps searching for food in a food patch before giving up and leaving the patch, in particular when habitat patches are small relative to the movement capacity of the species (Table 1). Individuals can vary in their behavioral type from exploratory ones with low ARS to less exploratory ones with high ARS [52]. To study such individual variation in ARS, every foraging patch encounter which results in ARS could be classified as a repeated measure and individual variation in ARS could be already assessed after a few days of data collection (depending on how many patches are encountered per day). When it comes to analyzing whether individuals differ in their plasticity towards the environment (Fig. 1b), the monitoring duration needed is obviously dependent on the temporal scales over which environmental conditions change. Importantly, the behavior of every individual should be measured repeatedly in each context (or over the environmental gradient) in order to account for individual variation in plasticity [98]. For example, variation in diel plasticity of lake depth use warrant repeated dive depth measures at day and night over several days for every individual [42], whereas questions related to e.g. seasonal variation in diel depth use warrant longer monitoring times, respectively [42]. Finally, if we are asking questions whether individuals differ in their behavioral development over age, or whether they differentially use (experiential or social) learning, (spatial) memory [127], exploration, and behavioral innovation [54, 127, 128, 133] to navigate in space we need long durations of monitoring, ideally over the entire lifespan of the animal.

**Table 1:** Examples of movement behaviors which have been shown to feature intra-population individual variation and temporal scales needed in order to quantify the behavior for analysis of between-individual repeatability. We roughly consider temporal resolutions of less than 1 fix per day to be “low”, one to three daily fixes to be medium, and bi-hourly or hourly fixes to be “high”. “Low” temporal resolution of movement data may also be achieved with visual resightings or DNA recaptures of individuals instead of biologging devices

Duration of monitoring	Temporal resolution	Example behavior	References
Long term (multi-annual, lifelong)	low	• breeding or stopover site selection and fidelity	[126]
		• migration probability, distance, and site fidelity	[50]
	medium - high	• movement and habitat selection in relation to age, memory, learning, behavioral innovation	[127–129]
Medium term (annual, seasonal)	low	• natal dispersal probability and distance	[130, 131]
	medium	• foraging site fidelity	[38]
	high	• resource selection	[44, 85, 86]
Short term (weeks, days)	low	• home range size	[52]
	medium	• daily net squared displacement	[132]
	high	• area restricted search behavior	[54, 58, 62]
		• diel activity	[27]

Last, movement data are often collected at different sampling rates across individuals and data may be collected from a mix of older and newer GPS tag models leading to differences in precision and error rates. Especially when tracing individual differences in movement, a careful cleaning of the data is indispensable to avoid the erroneous detection of individual differences caused by measurement error.

In addition to the practical requirements allowing us to measure behavior at the right temporal scale, there are important statistical considerations to be kept in mind. These primarily concern the number of individuals measured and the number of repeated behavioral measures per individual obtained. A meta-analysis has shown that the repeatability of behavioral traits, i.e. the proportion of total phenotypic variation in behavior explained by individual’s identity, is commonly relatively low (0.1–0.3 [80]). Simulations show well that reaching sufficient statistical power to estimate individual differences with high precision can be difficult when repeatability is low [134]. There are two ways in which the precision can be increased: either by increasing the number of individuals measured or by increasing the number of repeated measures per individual (see recommendations and supplementary material in [134]). The former may be limited by the costs per tracking unit, the difficulty to capture elusive species, and in case of threatened species ethical concerns with equipping many individuals with tracking devices, whereas the number of repeated measures is mainly limited by the battery lifetime of tracking devices. Most of the studies cited in this manuscript used 25–50 tracked individuals to assess individual variation.

## Worked example

Here, we provide an example of how repeatability, behavioral types, behavioral reaction norms, predictability, and behavioral syndromes can be estimated from movement data. In the Supplementary Material, we provide a full R tutorial and code through the statistical analysis of our worked example [51].

### Movement behavior of African elephants

**Fig. 3:**
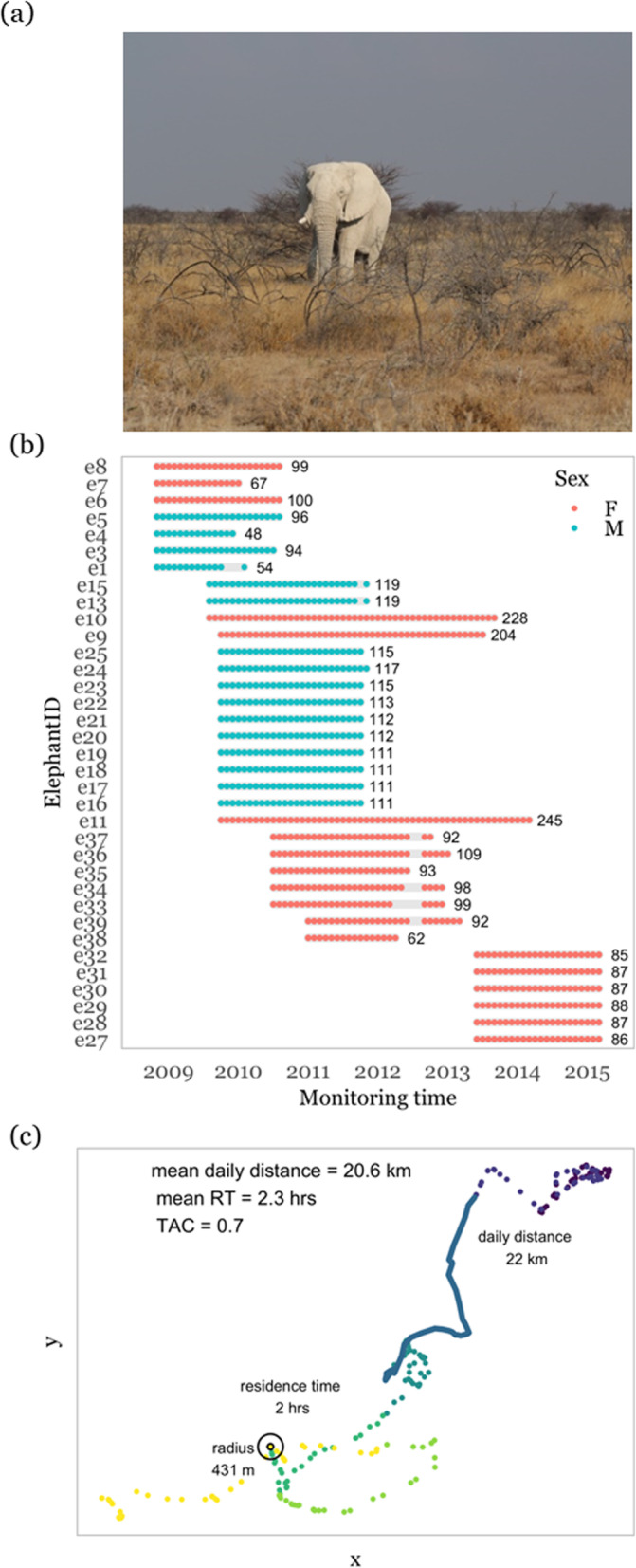
Sampling design used to study individual variation in African elephants (*Loxodonta africanus*, **a)** in Etosha Nationalpark. **b** Movement data were collected for 35 elephants between 2008 and 2015. Movement metrics were calculated on a weekly basis yielding 54–245 repeated measures per individual. **c** We divided the movement path of each elephant into weekly segments and calculated weekly means for three common movement metrics: daily movement distance, residence time (RT) in a circle of the individual’s average step length and turn angle correlation (TAC). We here show the movement path of one individual during a week in January

We use open access data (Movebank Data Repository [135, 136]) from 35 African elephants (*Loxodonta africanus*, Fig. 3a) which were monitored for at least 12 months (Fig. 3b). The data were previously published in Abrahms et al. [132] and Tsalyuk et al. [137]. Individual differences in movement behaviors were demonstrated for these elephants using a single measure per individual over its entire monitoring period [135]. Our methodological approach goes beyond that by repeatedly quantifying movement metrics for all individuals within discrete time steps, thereby allowing us to decompose variance into its among- and within-individual components, i.e. to estimate behavioral types and reversible plasticity, respectively, in trait expression. The detailed description of how the data was collected can be found in Tsalyuk et al. [137].

GPS fix intervals varied between 20 and 30 min and were resampled to 30 min for all individuals. From the movement tracks we calculated three common movement metrics on a weekly basis – mean daily movement distance, mean turn angle correlation, and mean residence time (Fig. 3c). Daily movement distance was calculated as the sum of 30-min displacement distances within 24 h. From this, mean daily movement distance was calculated as the mean of daily distances within one week. Longer mean daily movement distances are indicative of more active individuals which are travelling at faster speed, as compared to shorter mean daily movement distances. Turn angle autocorrelation was calculated as the inverse of angular autocorrelation [132], where angular autocorrelation was the sum of squares of chord distances between N successive turn angles within one week (ideally 336 steps) [138]. Individual variation in turn angle correlation could therefore be indicative of whether an individual’s movement is more or less exploratory [132]. Residence time was calculated as the number of hours an individual spent inside a circle of the radius of its mean step length centered on its GPS fix location [139]. We calculate the mean residence time over all locations within one week (ideally 336). Under spatial heterogeneity of resource availability, a higher mean residence time could be indicative of higher resource availability in this given area as compared to an area occupied by an individual with a lower mean residence time. If spatial heterogeneity is controlled for (see section “How to disentangle intrinsic individual variation from reversible behavioral plasticity” above) then individual variation in mean residence time could be interpreted as variation in exploration behavior. Turn angle correlation and residence time were calculated with code published in Abrahms et al. [132]. We obtained between 54 and 245 weekly behavioral measures for each elephant (Fig. 3b). Our worked example represents an example where we quantify individual variation in tracking data with a medium long monitoring with fairly high temporal resolution (see Table 1).

### Statistical methods

Generalized linear mixed model (GLMMs) provide a powerful tool to decompose observed behavioral variation into environmental fixed effects and within- and between-individual components [30].

First, normal mixed effects models, with a random intercept for individual, allow to estimate whether individuals differ in their behavioral type for a behavior (the individual level predictors of the random intercept, Fig. 1a) by quantifying repeatability. We fit mixed effects models separately for weekly means of daily movement distance, turn angle correlation and residence time. We controlled for sex differences (two-level factor) and accounted for population level shifts in behavior over the course of the year (using month as a continuous covariate with a second order polynomial). We also included a random intercept for month nested in year to account for measures taken within a given month of a given year being more similar to each other. Finally, we included a random intercept for the individual.

Second, random regression models allow to estimate reaction norm components (i.e. the individual level random intercept, the random slope over an environmental gradient, and their correlation), giving insights into a) whether individuals differ in behavioral type (the random intercept), b) whether individuals differ in behavioral plasticity along environmental gradients (the random slope) and, c) whether plasticity depends on the behavioral type (intercept and slope correlation, Fig. 1b). We here fit a random regression model for mean daily movement distance to uncover individual variation in movement plasticity towards seasonal variation in the environment using month of the year as a surrogate variable [137]. Additionally to the random intercept for the individual we therefore also included non-linear random slopes for study month. We fitted mixed models within a Bayesian approach using brms [140] and compared model fit using the widely applicable information criterion (WAIC, [141]).

Third, extending random regression models to a double-hierarchical model (DHGLM) allows us to simultaneously model individual variation in intercept (behavioral type), slope (behavioral plasticity), residual variance (predictability), and their correlations [94–96]. High residual variance is indicative of low predictability, whereas low residual variance indicates high predictability (Fig. 1c). Additionally to the variance structure on the response variable mean daily distance, we also imposed a variance structure on the residual variance, partitioning this residual variance per individual. For modelling purposes, individual variation in within-individual variance is estimated on the log scale, for biological interpretation we backtransformed individual values to the original km scale. We calculated the coefficient of variation in predictability (CV_P_) [94].

Finally, multivariate mixed effects models allow to estimate whether two (or more) behaviors are correlated strictly at the among-individual level of variation (i.e. correlation of the mean trait values; behavioral syndromes) or within-individual level of variation (i.e. correlated plasticity) (Fig. 1d, [30, 84]). Using brms [140], we fit a multivariate mixed effects model with mean daily distance, residence time, and turn angle correlation as response variables. We controlled for a non-linear effect of month and for sex as fixed effects and for random intercepts for the individual. The model produces the among-individual correlations among the three behaviors.

### Components of individual variation in the movement of African elephants

**Fig. 4:**
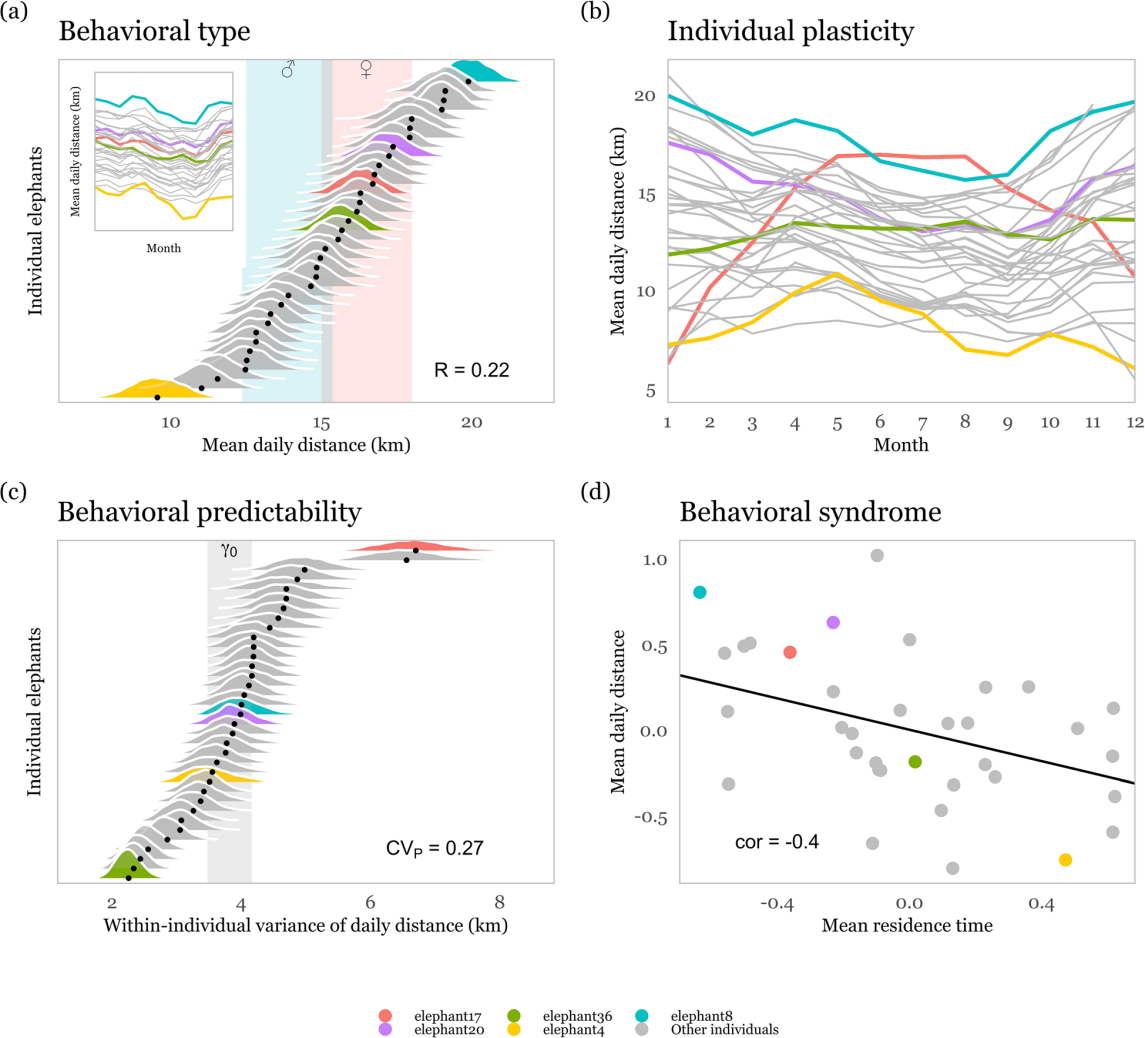
Individual variation in the movement behavior of 35 African elephants. We highlight five individuals (elephant 4, 8, 17, 20, 36) to facilitate the interpretation of individual differences across hierarchical levels: **a**) Elephants differed in their average behavioral type for daily movement distance from individuals with a daily movement distance of 10 km (elephant 4) to 20 km (elephant 8) in January. Shaded areas indicate the posterior 95% credible interval of the population level daily movement distance for male (blue) and female (red) elephants, respectively. 22% of the variation in daily movement distance was due to differences among individuals. Individual variation in behavioral shifts over time are unaccounted for (inset) (**b**) Elephants however differed in how they adjusted their movement behavior over month of the year. Most elephant decreased movement from the beginning of the year (wet season) towards the middle of the year (dry season) and then increased movement again towards the end of the year. Some elephants however did not adjust movement (elephant 36) or increased movement (elephant 17) during the middle of the year. **c** Elephants differed in within-individual variance from less predictable individuals (e.g. elephant 17) with high variance around their behavioral mean to more predictable individuals (e.g. elephant 36) with low variance around their behavioral mean. The posterior 95% credible interval of the population level residual variance (γ_0_) is shown in gray. Note, we here show exponentiated model estimates to facilitate biological interpretation on the km scale. **d** Elephants with on average longer daily movement distances also had on average shorter residence times. The among-individual correlation between the two behaviors was − 0.4. Because distance (km) and residence time (hrs) are on different scales, behaviors were scaled to a mean of 0 and standard deviation of 1 prior to model fitting. Figures are based on bayesian model results

In our worked example we indeed found that, over the course of 1–4.5 years, elephants differed in three basic parameters of their movement strategy. While controlling for simple seasonal changes in behavior and sex differences, 22% of the variation in daily movement distance and 17 and 30% of the variation in residence time and turn angle correlation, respectively, could be attributed to individual differences [posterior mean ± 95% credible interval R_meanDailyDistance_ = 0.22 [0.18, 0.27], R_meanRT_ = 0.17 [0.13, 0.22], R_TAC_ = 0.3 [0.26, 0.35]). Behavioral types for e.g. daily movement distance ranged from elephants which moved on average 9.5 km a day to elephants which moved on average 19 km (Fig. 4a). It is important to keep in mind that other factors that were not include in this worked example, such as differences in the habitat experienced by individual elephants, differences in age, or in social group composition may explain parts of these individual differences. The intention of this example is to be a “how to” guide for ecologists. For a research example that attempts to draw actual conclusions on individual variation in elephant behavior one would need to include more covariates. Elephants also differed in how they changed their behavior over the temporal gradient month of the year, which captured seasonal variation in temperature, rain, and forage availability (Fig. 4b). Compared to the random intercept model (WAIC = 21,749), the random intercept and slope model (WAIC = 21,224) produced a better fit for the data (∆WAIC = 524). While most elephants reduced their movement distance during June to October (the drier months of the year e.g. blue and purple reaction norm lines in Fig. 4b), the magnitude of movement decrease varied among individuals and some elephants did not reduce movement at all (green line) or even increased movement (red line) during June to October. After controlling for individual variation in movement type and in seasonal adjustments of movement, elephants still differed in within-individual variability, i.e. in the amount of their unexplained behavioral variance (CV_P_._meanDailyDistance_ = 0.27 [0.2, 0.35]). Within-individual variance ranged from 2.25 to 6.7 km (Fig. 4c), indicating that some individuals were much more predictable in their behavior than other elephants in the population. In addition, we found among-individual correlations between some variance components. First of all we did not find a correlation between behavioral type and behavioral plasticity (cor_int.month_ = − 0.02 [− 0.36, 0.34], cor_int.month_^2^ = 0.26 [− 0.07, 0.56]), i.e. elephants with a higher average daily movement distance did not systematically change their behavior more (or less) strongly over month of the year than elephants with a lower average daily movement distance (Fig. 3b). However, behavioral type was correlated with within-individual variability (cor = 0.5 [0.15, 0.74]) such that elephants that moved over longer daily distances were also less predictable. Last, we found a behavioral syndrome where individuals that moved on average over shorter daily distances also had on average longer residence times (cor = − 0.41 [− 0.68, − 0.1], Fig. 4d), whilst turn angle correlation was uncorrelated with these movement traits at the individual level.

We here present an example on how repeated measures of movement metrics can be used to analyze individual variation in the average, plasticity, and predictability of such movement metrics and their correlations among individuals. Tsalyuk et al. [137] previously demonstrated for this population that individuals differed in their movement directedness (analogous to TAC in our analysis) and movement speed (analogues to movement distance in our analysis). Our analysis complements these earlier findings by demonstrating long-lasting individual differences in the average expression of these behaviors. More importantly our approach shows that individual elephants differ in how they adjust movement over the course of the year and in how predictable they are in their movement tactics. Male elephants generally moved over shorter daily distances and were less predictable in their daily movement distance than female elephants. Two of our movement metrics – movement distance and residence time – were correlated into a movement behavioral syndrome. While one might argue that these movement traits are not independent and do not reflect functionally distinct movement behaviors, evaluating the stability of such syndromes along environmental gradients may be particularly interesting [46]. Using a different statistical approach, Bastille-Rousseau and Wittemyer [86] recently demonstrated, for a population of African elephants in Kenya, that individuals showed long-term consistency for nine distinct habitat selection tactics. Similar to behavioral syndromes, these tactics combined selection for several spatial characteristics, like landcover, water or poaching risk. Tactics were spatially segregated, indicating that habitat composition may be an important driver for the emergence of different selection tactics. All the more surprising, elephants that were closely related and spent a significant amount of time together (i.e. in the same herd) showed distinctly different selection tactics, a convincing case that intrinsic individual differences caused by non-genetic factors, such as experiential learning, also contribute to the emergence of these different tactics. As a concluding remark we would like to highlight that elephants have a remarkable movement capacity and indeed range over large areas in Etosha NP, as a worked example to demonstrate a conceptual framework we did not control for the effects of local vegetation and habitat composition on movement types and their plasticity. Future empirical studies could use a variance partitioning approach to test how individual elephant behavioral types change movement in relation to human infrastructure or agriculture or whether certain behavioral types are particularly vulnerable to selection by poachers (similar to [86]).

## Discussion and outlook

We showed how variance partitioning approaches developed in behavioral ecology can be brought to movement ecology and applied to movement data to study not only reversible but also intrinsic variation among individuals. We highlighted the three different forms of among-individual variation formulated in behavioral ecology and their covariance and reviewed the current evidence for such variation in movement behavior. We also discussed ways to disentangle intrinsic individual variation from behavioral plasticity and the inherent limitations to control for all relevant aspects of the environment in the wild. Studying among-individual variation in movement can facilitate ecologically and evolutionary meaningful research. For example the POLS hypothesis (see paragraph on behavioral syndromes) predicts that individuals within populations differ in suites of traits along a fast-slow continuum where fast individuals exhibit faster growth rates and invest in early reproduction at the cost of lower survival as compared to slow individuals [106]. The lower survival of individuals investing into early reproduction is assumed to be mediated by the expression of risk enhancing behaviors which facilitate resource acquisition at the expense of survival [106, 142, 143]. Individual variation in movement may therefore translate into individual variation in resource acquisition, body mass, reproductive output per attempt, and survival [143]. Another example concerns how animal populations will be able to adapt to landscape change. On a global scale, landscape fragmentation and anthropogenic features have recently been shown to restrict animal movements (on a population level) across terrestrial mammals [144]. However, behavioral ecologists predict that individuals may differ in their ability to cope with landscape change and hence how they move through these landscapes; some individuals are expected to move more easily through our modernized landscape [69]. They may for example be quicker to use anthropogenic features developed to aid connectivity, like road crossing structures [145–147]. Variation is the key ingredient for selection and evolution and recent evidence for the heritability of movement traits [101] suggests that behaviorally diverse animal populations have the potential to adapt to the challenges of the Anthropocene within a few generations. Movement data is ideal for testing such predictions in elusive wildlife by adopting the theory and statistical tools (i.e. variance partitioning) from behavioral ecology and quantitative genetics [148, 149]. This would bind movement ecology more tightly into an evolutionary ecological framework.

Scientists may find valuable biological meaning in patterns of individual variation. In other cases, scanning data for the presence or absence of individual variation may merely be a step of data exploration either for validation of statistical assumptions, for supporting model simplification, or for corroborating population level mean effect conclusions. We recommend that estimating and studying individual differences in movement behavior should be an integral part of data analysis in movement studies.

Increasing amounts of movement data from various taxa and species are being collected and deposited into standardized databases, like the Movebank Data Repository (https://www.movebank.org/) or EUROMAMMALS (http://www.euromammals.org) to promote collaborative science examining general patterns beyond local populations. This offers unique opportunities to study the extent of individual variation in movement behavior across ecosystems and species. Recognizing individuals with specific movement patters, and how sensitive they are to environmental variation, might further be valuable, for example, when making decisions related to animal conservation. Certain behavioral types may for example be better suited for animal translocations, or cope better with landscape fragmentation and urbanization.

## Conclusions

We here show that movement data are a promising data source to reveal individual differences in the behavior of wildlife. To date this individual variation is however rarely systematically analyzed or even considered as biologically interesting phenomenon. Using statistical tools from the modern behavioral ecology literature we demonstrate how partitioning behavioral variance into its among- and within-individual sources can give new insights about the biology hidden behind the population mean trait expression. Individual differences in movement are important because it means that individuals differ in how they move through the landscape and their likelihood to encounter conspecifics, prey or landscape features. These may have important consequences for ecology and evolution of movement behaviors. Ignoring individual differences in movement, while solely interpreting population level effects, may in the worst case misrepresent true underlying mechanisms in how animals move.

## Supplementary information

**Additional file 1.** Full R code and tutorial guiding through the statistical methodology of the worked example.

**Additional file 2.** Movement metrics of 35 African elephants used to study individual variation in movement (input for Additional file 1).

## Data Availability

A complete R tutorial for reproducing the statistical analysis in this manuscript is provided in Additional file 1. The tutorial includes pre-processed monthly estimates of movement metrics. The primary GPS data for African elephants were partially published in the Movebank Data Repository with DOI 10.5441/001/1.hm5nk220 and 10.5441/001/1.3nj3qj45.

## Abbreviations

ARS: Area restricted search behavior
BLUP: Best liner unbiased predictor
POLS: Pace of life syndrome
TAC: Turn angle correlation
