# Supplementary material for: A guide for studying among-individual behavioral variation from movement data in the wild
Source: Mov Ecol. 2020 Jun 29;8:30. doi: 10.1186/s40462-020-00216-8 (PMC7325061; doi:10.1186/s40462-020-00216-8)
Supplement: Supplementary file 1 — Additional file 1. Full R code and tutorial guiding through the statistical methodology of the worked example. [file 40462_2020_216_MOESM1_ESM.pdf]

Tutorial: A guide for studying among-individual behavioral variation from movement data in the wild.

Anne G. Hertel & Petri T. Niemelä

June 09, 2020

Contents

Aim 1

Elephant movement data 2

Behavioral types and repeatability: Mixed modelling approaches 5
- Frequentist approach . . . 5
- Bayesian Approach . . . 14

Variation in behavioral plasticity: Random regression models 18
- Frequentist approach . . . 18
- Bayesian approach . . . 21

Variation in behavioral predictability: Double hierarchical mixed effects models 24
- Correlation between intra-individual variability and behavioral types . . . 30

Behavioral syndromes: Multivariate mixed effects models 31

Concluding remarks 35

Author affiliation and Funding 36

Acknowledgements 36

Aim

This tutorial accompanies our 2020 Movement Ecology paper "A guide for studying among-individual behavioral variation from movement data in the wild.". The aim of this tutorial is to illustrate how simple univariate mixed models can be used to partition behavioral variance into its among - and within individual sources. We will then go on to illustrate how we can fit univariate mixed models with random slopes to estimate individual variation in behavioral plasticity. Third, we will demonstrate how to test whether individuals differ in intra-individual variability. Finally, we introduce multivariate mixed models with multiple response variables to estimate among-individual level correlation of behaviors. We will cover four principal concepts from the animal personality literature: a. Variation in behavioral types and repeatability b. Variation in behavioral plasticity c. Variation in behavioral predictability d. Behavioral syndromes. With this tutorial we hope to offer a suite of easy pathways into analyzing individual variation in movement more explicitly to expand the biological questions movement ecologists can seek answers to.

We further would like to refer the interested reader to a seminal paper in the field: Dingemanse & Dochtermann (2013) "Quantifying individual variation in behavior: mixed effects modelling approaches." Journal of Animal

Ecology, 82, 39-54. doi: 10.1111/1365-2656.12013.

The here presented tutorial was inspired by a series of tutorials by Tom Houslay who has synthesized analyzing animal personalities and behavioral syndromes with the R package MCMCglmm in a very user friendly way (https://tomhouslay.com/tutorials/). Equivalent to MCMCglmm we here will introduce and use the brms package which allows the application of double hierarchical mixed effects models, needed to estimate individual variation in predictability:

Bürkner (2017) "brms: An R Package for Bayesian Multilevel Models Using Stan." Journal of Statistical Software, 80(1), 1–28. doi: 10.18637/jss.v080.i01.

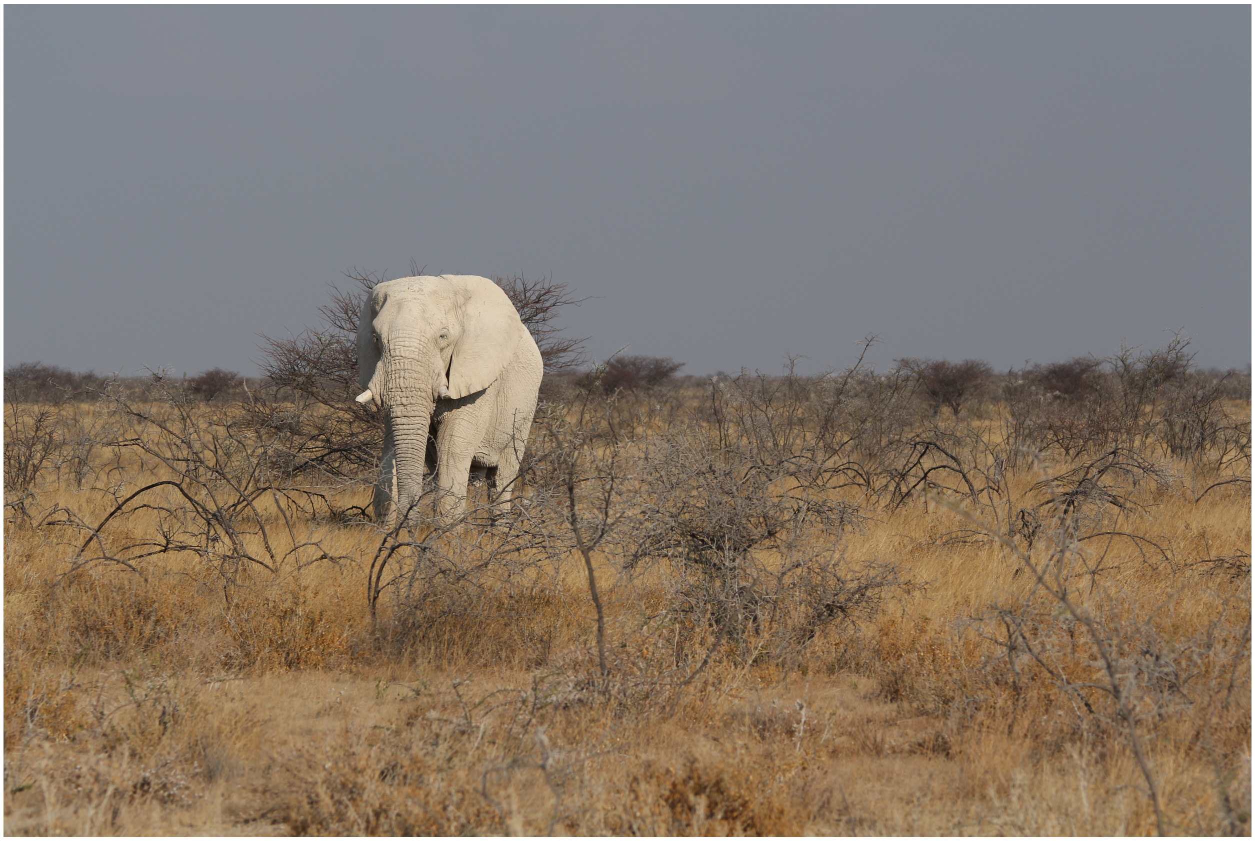

Figure 1: African elephant (*Loxodonta africanus*)

Elephant movement data

Here we use movement data from 35 African elephants (*Loxodonta africana*) monitored in Etosha Nationalpark, Namibia to estimate individual variation in patterns of movement.
The raw data had a temporal resolution of 30 minutes, from which we quantified a set of common movement metrics for each elephant. We quantified metrics on a weekly basis and to avoid bias introduced by missing locations, included only weeks with at least 200 successful fixes (out of 336 possible).

We quantified:

- mean daily movement distance (km)
- turn angle correlation (tac)
- mean residence time (meanRT)

The data we use for the purpose of this tutorial was previously published in:

Tsalyuk M, Kilian W, Reineking B, Marcus W (2019) Temporal variation in resource selection of African elephants follows long term variability in resource availability. Ecological Monographs. doi:10.1002/ecm.1348.

and

Abrahms B, Seidel DP, Dougherty E, Hazen EL, Bograd SJ, Wilson AM, McNutt JW, Costa DP, Blake S, Brashares JS, Getz WM (2017) "Suite of simple metrics reveals common movement syndromes across vertebrate taxa." Movement Ecology 5:12. doi:10.1186/s40462-017-0104-2

and are publicly available under

Kilian W, Getz WM, Zidon R, Tsalyuk M (2018) Data from: Temporal variation in resource selection of African elephants follows long term variability in resource availability. Movebank Data Repository. doi:10.5441/001/1.3nj3qj45

and

Abrahms B (2017) Data from: Suite of simple metrics reveals common movement syndromes across vertebrate taxa. Movebank Data Repository. doi:10.5441/001/1.hm5nk220

We first install and load a suite of R packages.

```
library(lme4);library(arm);library(MuMIn);library(tidyverse)
library(plyr);library(broom);library(coda);library(grid)
library(gridExtra);library(brms); library(broom.mixed); library(merTools);
library(tidybayes);library(parallel)

# extract legend function
g_legend<-function(a.gplot){
  tmp <- ggplot_gtable(ggplot_build(a.gplot))
  leg <- which(sapply(tmp$grobs, function(x) x$name) == "guide-box")
  legend <- tmp$grobs[[leg]]
  return(legend)}

data<-readRDS("mov.metrics_weekly.rds")
head(data, n = 3)
```

```
##   animal_id             Week_id meanDailyDisplacement   meanRT       tac week
## 1 elephant1  elephant.1_0_1_2009              26.77996 5.804233 0.8028475    0
## 2 elephant1  elephant.1_1_1_2009              25.96528 2.755988 0.7602063    1
## 3 elephant1 elephant.1_10_3_2009              21.68015 1.856716 0.9053003   10
##   month year Sex
## 1     1 2009   M
## 2     1 2009   M
## 3     3 2009   M
```

Mean daily displacement reflects the average daily distance (in kilometers) an elephant moved during a given week. Mean residence time reflects the mean time (in hours) an elephant spend within a circle of its mean step length during that week. And finally a high turn angle correlation (tac) indicates that movements are centered within a certain area whereas a low tac indicates more exploratory behavior during that week. Next to the behavioral metrics this data frame contains information about the individual (animal_id), the week, month, and year in which the behavior was measured (month, year), and the sex of the individual.

```
tail(table(paste(data$animal_id,data$year,sep="_"),
      data$month))
```

```
##
##                    1  2  3  4  5  6  7  8  9  10  11  12
```

```
##   elephant8_2010 5 4 5 5 4 5 5 2 0  0  0  0
##   elephant9_2009 0 0 0 0 0 0 0 0 0  1  4  4
##   elephant9_2010 5 4 5 4 4 5 5 4 5  5  4  5
##   elephant9_2011 4 4 5 5 4 5 5 5 5  4  5  5
##   elephant9_2012 4 5 5 4 5 5 4 5 4  5  5  3
##   elephant9_2013 5 5 5 4 5 4 2 0 0  0  0  0
```

```
tail(table(data$animal_id,data$month))
```

```
##
##                1  2  3  4  5  6  7  8  9 10 11 12
##   elephant4  5  1  3  3  3  4  3  4  5  5  5  7
##   elephant5 10  8  8  8  8  9 10  7  5  5  8 10
##   elephant6 10  8  9 10  9  9 10  7  5  5  8 10
##   elephant7  8  4  4  5  5  4  5  4  5  5  8 10
##   elephant8 10  8  9 10  9  9 10  6  5  5  8 10
##   elephant9 18 18 20 17 18 19 16 14 14 15 18 17
```

We have data for 35 individual elephants monitored for a minimum period of one year (range 1 - 6 years). Behaviors were assessed weekly, hence an individual was annually assessed for 1 - 5 weeks per month. Across multiple monitoring years, the number of monthly repeats ranged between 1 and 24. …

```
table(data$year)
```

```
##
## 2008 2009 2010 2011 2012 2013 2014 2015
##   60  580 1086 1030  343  293  302   72
```

… and these elephants where monitored over eight years between 2008 and 2015.

Visual data exploration suggests that elephants shift their behavior over the course of the year (Fig 2). Elephants move least between June and October, the dry season in Etosha. At the same time Turn angle correlation (tac) is highest. Mean residence time seems largely unaffected by seasonality.

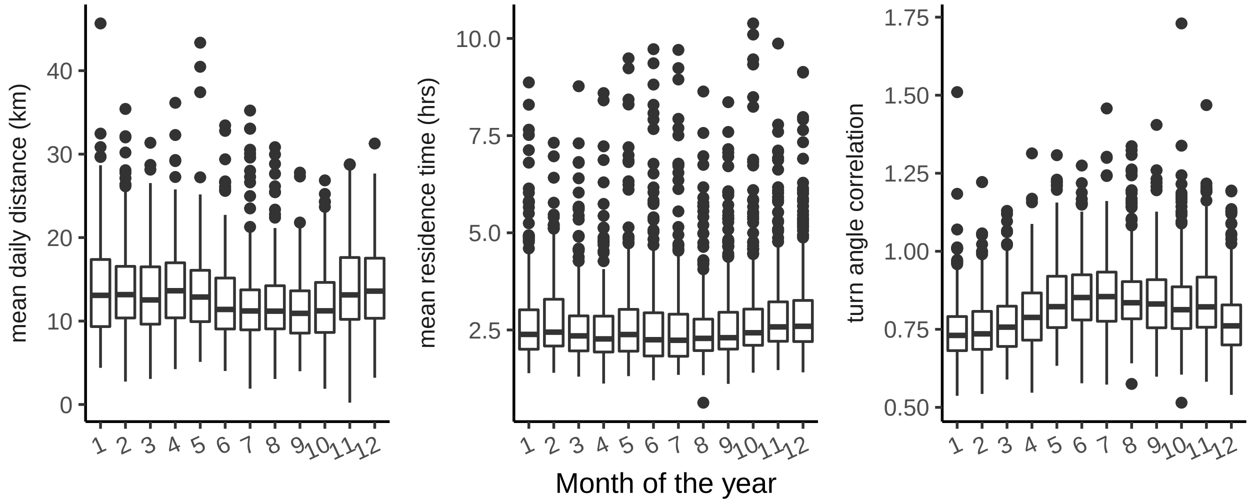

Figure 2: Data exploration: Movement metrics over month of the year

Female elephants seem to move over longer daily distances, have longer residence times and higher turn angle correlation than males (Fig 3).

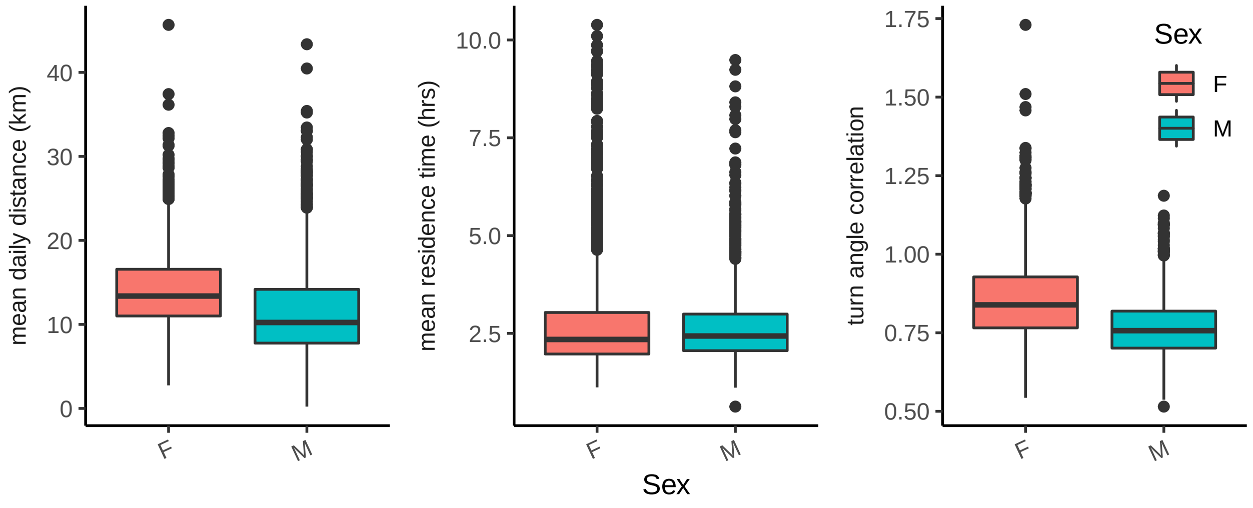

Figure 3: Data exploration: Sex effects on movement metrics

Finally, when just *visually* exploring data for e.g. the month of February, we still see that there is quite a lot of variation among individuals (Fig 4), whether these individual differences persist over the time span where the data is collected, remains to be seen.

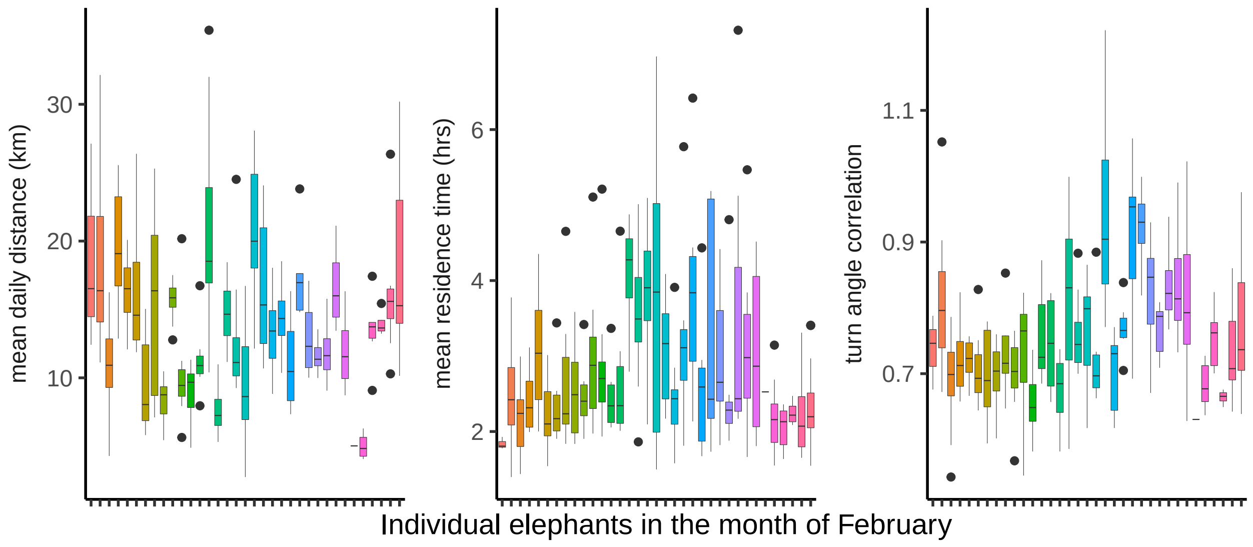

Figure 4: Data exploration: Individual variation in February

Behavioral types and repeatability: Mixed modelling approaches

Frequentist approach

We use the R package lme4 to fit a mixed model on one of the behaviors. We control for behavioral shifts over the course of the year by including month (1:12) as a fixed effect. "Month"" is a time variable which stands respectively for seasonally changing environmental variables, which are temporally autocorrelated, like e.g. temperature, precipitation and resulting food availability. Conditions in a given month (e.g. July) are

more similar to the preceding and following months (e.g. June or August) than to months that are farther away in time. Because of the cyclic nature of "month of the year" (our month coding starts in January - "1" and ends in December - "12") we need to include month as a non-linear term which allows predictions at the start and end of the year to be more similar to each other. We chose a simple second order polynomial term. We also control for behavioral differences caused by sex as fixed effect. Mixed models allow us to determine how much of the remaining behavioral variation is due to differences among individuals. We will also add a random effect for study year (2008 - 2015).

Although we expect the reader to be familiar with mixed models - here a short recap: In a mixed model we estimate how fixed effects - like month or Sex - explain variation in behavior. We additionally assume that measurement points taken from the same individual (or year) are more similar to each other than expected by random, i.e. they are not independent. By fitting a random intercept for individual identity we allow the mean behavioral expression (i.e. intercept) to vary among individuals.

Behavioral types and repeatability of daily movement distance

```
m1 <- lmer(meanDailyDisplacement ~ month + I(month^2)  + Sex +
             (1|animal_id) + (1|year/month), data)
par(mar=c(2, 2, 2, 2));qqnorm(residuals(m1),main=NULL)
```

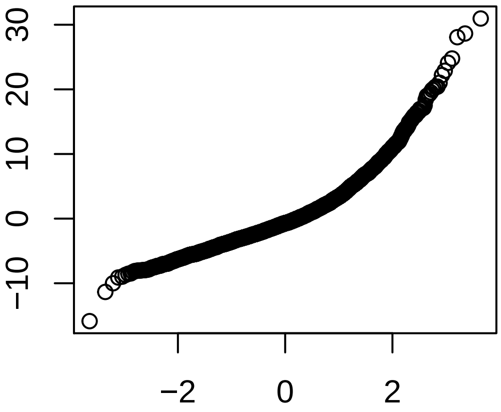

Figure 5: Q-Q Plot to inspect normality of residuals.

```
summary(m1)
```

```
## Linear mixed model fit by REML ['lmerMod']
## Formula: meanDailyDisplacement ~ month + I(month^2) + Sex + (1 | animal_id) +
##     (1 | year/month)
##    Data: data
##
## REML criterion at convergence: 21878.4
##
## Scaled residuals:
##     Min      1Q  Median      3Q     Max
## -3.6969 -0.6292 -0.1656  0.4174  7.2153
##
## Random effects:
##  Groups     Name        Variance Std.Dev.
##  month:year (Intercept)  1.0634  1.0312
##  animal_id  (Intercept)  5.0225  2.2411
##  year       (Intercept)  0.2253  0.4746
```

```
##  Residual              18.4111  4.2908
## Number of obs: 3766, groups:  month:year, 77; animal_id, 35; year, 8
##
## Fixed effects:
##             Estimate Std. Error t value
## (Intercept) 16.48670    0.73676  22.377
## month       -0.95351    0.17997  -5.298
## I(month^2)   0.06741    0.01349   4.997
## SexM        -2.57661    0.78637  -3.277
##
## Correlation of Fixed Effects:
##            (Intr) month  I(m^2)
## month      -0.595
## I(month^2)  0.527 -0.974
## SexM       -0.475 -0.003  0.005
```

```r
r.squaredGLMM(m1)
```

```
##             R2m      R2c
## [1,] 0.08483256 0.318461
```

As expected, at the population level, both the time of year and the sex of the individual affect how far elephants move over the course of a day (Fig 6).

```r
ggplot(data,
       aes(x = month, y = meanDailyDisplacement, color = Sex)) +
  geom_jitter(size=0.5) +
  geom_line(aes(y = predict(m1,re.form = NA)),size=1.5) +
  theme_classic()+
  ylab("mean daily distance (km)")+
  scale_x_continuous(breaks = seq(1,12,1))
```

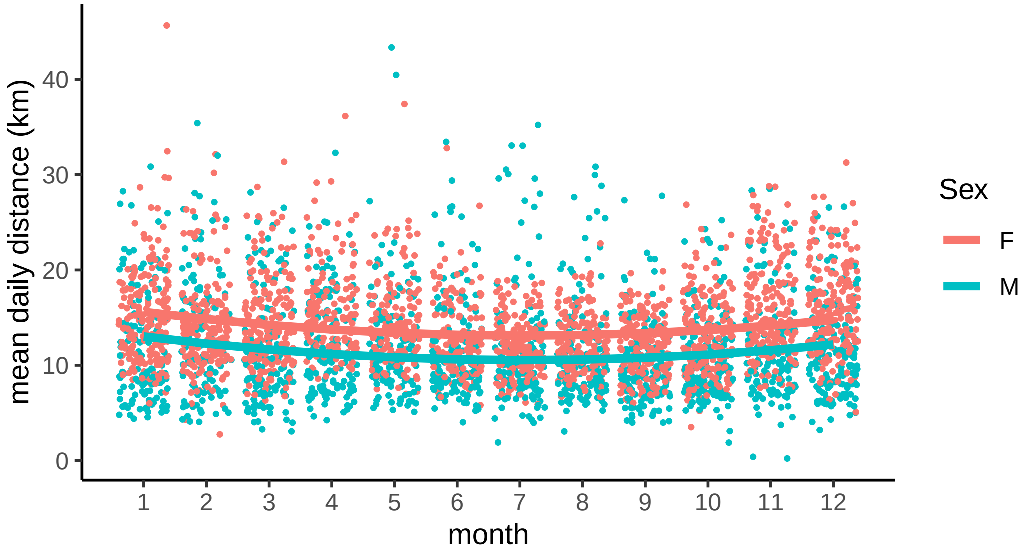

Figure 6: Population level shift in daily movement distance over the course of the year (January - December) for female (red) and male (blue) African elephants.

Below we show you the prediction lines for each individual and year combination (Fig 7). As you can see - some individuals are predicted to move consistently less and others are predicted to move consistently more.

We did not test here whether individuals differ in how they move over the course of the year, hence the slope over month is the same for all individuals (but see the section on reaction norms).

```
pr <- data.frame(animal_id = data$animal_id,
                 month = data$month,
                 year = data$year)
pr$fit <- predict(m1)
pr$EleYear <- paste0(pr$animal_id,pr$year)

ggplot() +
  geom_line(data = pr,
            aes(x = month,
                y = pr[,"fit"],
                group = pr$EleYear,
                color = pr$animal_id), size=0.3)+
  labs(y = "Predicted daily distance (km)",color = "ElephantID")+
  theme_classic()+
  theme(legend.position="none")+
  guides(color = guide_legend(nrow = 4))+
  scale_x_continuous(breaks = seq(1,12,1))
```

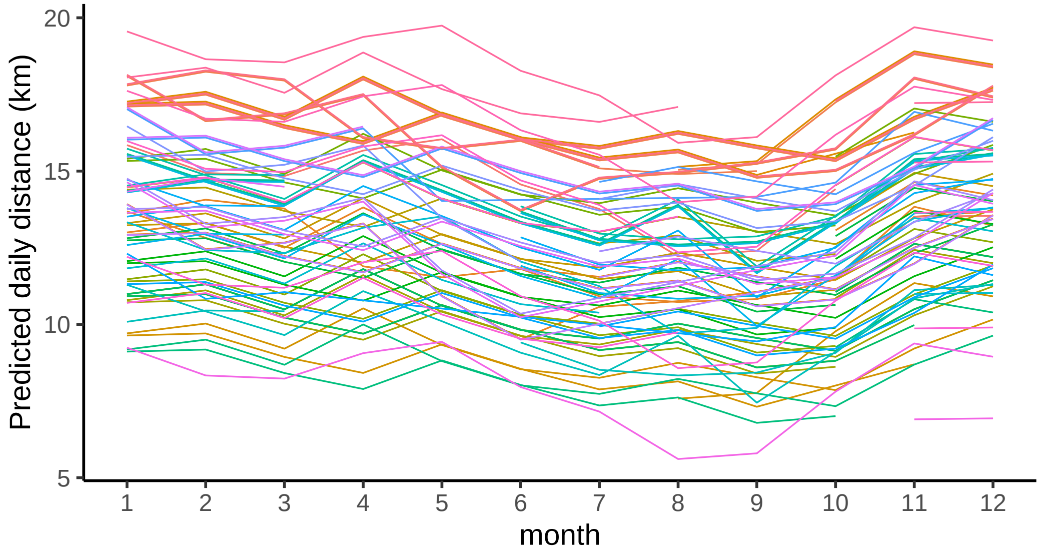

Figure 7: Individual differences in daily movement distance over the course of the year (January - December) for 35 African elephants.

It is always good practice to check whether model predictions actually match the observed data (Fig 8). Here we plotted prediction lines for five elephants and their corresponding observed movement distances. We can visually confirm that our predictions match the data quite well for most elephants. Elephant 17 however increases movement in June - August which is not reflected by the population level shift of movement over month. We will come back to this in the section on behavioral reaction norms below.

For consistency and to facilitate interpretation we will keep highlighting the same five individuals throughout the remainder of the tutorial.

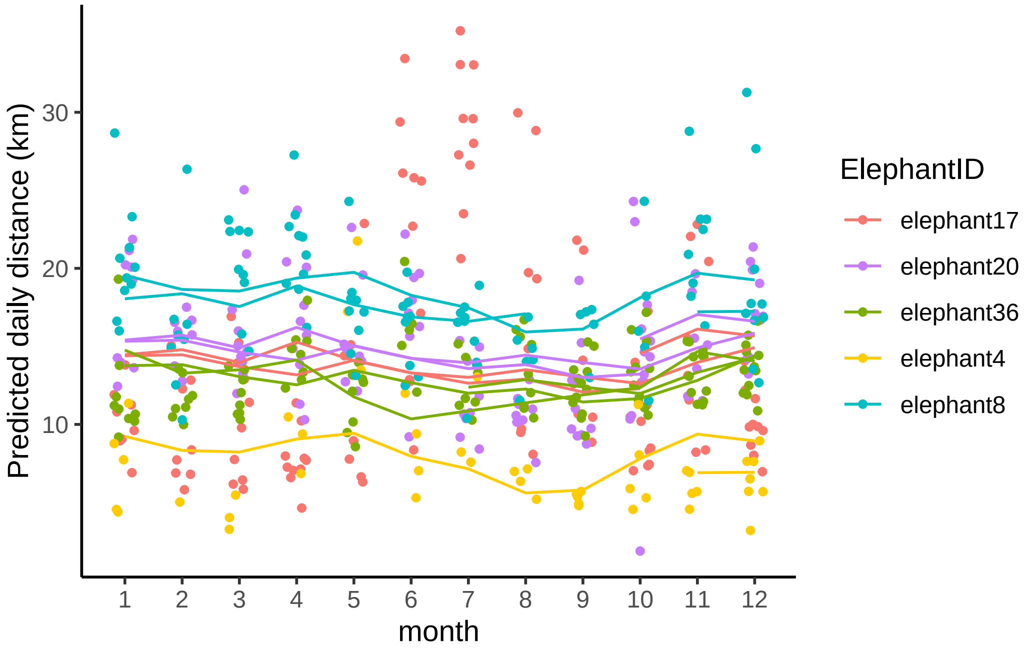

Figure 8: Prediction lines for daily movement distance in 5 African elephants and their observed movement distance.

We can now calculate repeatability i.e. variance standardized individual variation in focal behavior. We divide the variance explained by animal_id by the total phenotypic variance (animal_id + month:year + year + residual variance).

```
print(VarCorr(m1),comp = c("Variance","Std.Dev."))
```

```
##  Groups     Name        Variance Std.Dev.
##  month:year (Intercept)  1.06341 1.03122
##  animal_id  (Intercept)  5.02254 2.24110
##  year       (Intercept)  0.22528 0.47463
##  Residual               18.41106 4.29081
```

```
5.02255 / (5.02255 + 1.06342 + 0.22525 + 18.41106)
```

```
## [1] 0.2031589
```

```
VarCorr(m1)$"animal_id"[1] /
  (VarCorr(m1)$"animal_id"[1] +
     VarCorr(m1)$"month:year"[1] +
     VarCorr(m1)$"year"[1] +
     attr(VarCorr(m1), "sc")^2)
```

```
## [1] 0.2031585
```

In the same fashion we can calculate whether there is among-month within a given year (consistent variation in the month of January among the study years 2008 - 2015) and consistent among-year variation, i.e. if all elephants move consistently more or less in some years (resp. months) as compared to others. In fact elephants do not seem to show consistent behavioral variation between years (i.e. year identity explains ~1% of the variation in the focal behavior) or months nested in years (~ 4% of variance). Unless we a-priori expected that study year should have a strong effect on behavior (e.g. due to strong inter-annual variation in food availability) we may even consider to simplify our model by removing the random intercept for year.

```
VarCorr(m1)$"month:year"[1] /
  (VarCorr(m1)$"animal_id"[1] +
```

```r
    VarCorr(m1)$"month:year"[1] +
    VarCorr(m1)$"year"[1] +
    attr(VarCorr(m1), "sc")^2)
```

```
## [1] 0.04301409
```

```r
VarCorr(m1)$"year"[1] /
  (VarCorr(m1)$"animal_id"[1] +
    VarCorr(m1)$"month:year"[1] +
    VarCorr(m1)$"year"[1] +
    attr(VarCorr(m1), "sc")^2)
```

```
## [1] 0.009112348
```

The repeatability value ranges between 0 and 1 and indicates the proportion of variance explained by a given random effect, after controlling the model for the fixed effects of month and sex in our case. The repeatability value by itself however does not come with a measure of uncertainty. We can however simulate our model 1000 times in order to get a posterior distribution for all variance components, which we can use to calculate credible intervals and infer significance.

```r
set.seed(1)
simulated <- sim(m1, n.sim = 1000)

posterior_animal_id <- apply(simulated@ranef$"animal_id"[ , , 1],1,var)
posterior_yearmonth <- apply(simulated@ranef$"month:year"[ , , 1],1,var)
posterior_year      <- apply(simulated@ranef$"year"[ , , 1],1,var)
posterior_residual  <- simulated@sigma^2

quantile(posterior_animal_id /
        (posterior_animal_id + posterior_yearmonth + posterior_year + posterior_residual),
         prob=c(0.025, 0.5, 0.975))
```

```
##      2.5%       50%     97.5%
## 0.1756550 0.2186341 0.2680693
```

```r
quantile(posterior_year /
        (posterior_animal_id + posterior_yearmonth + posterior_year + posterior_residual),
         prob=c(0.025, 0.5, 0.975))
```

```
##        2.5%         50%       97.5%
## 0.003138401 0.004970995 0.008397671
```

```r
quantile(posterior_yearmonth /
        (posterior_animal_id + posterior_yearmonth + posterior_year + posterior_residual),
         prob=c(0.025, 0.5, 0.975))
```

```
##       2.5%        50%      97.5%
## 0.03182955 0.04256004 0.05523531
```

As we can see the credible interval around our repeatability estimate for individual variation is relatively wide (0.18 - 0.27). A reason for this could be that the number of individuals tested is still relatively limited (n = 35).

We can see that on average 22% of the remaining variance (after controlling for month and sex) in daily movement distance can be attributed to differences between individuals. This means that some elephants always move over shorter distances compared to other elephants and this difference is not caused by predictable monthly variation or sex differences. In a real study we would probably fit other relevant explanatory variables, like age of the animal, herd identity, and habitat composition in the elephants monthly range. On the other hand there does not seem to be a year effect on movement with elephants moving more or less in a particular

year (R.year = 0.004).

Alternatively to repeatability we can also calculate the coefficient of variation for between individual variance (CV_i). CV_i is not confounded by within-individual effects and is hence a better measure to compare the extent of between-individual differences among populations and traits. It is calculated as

$CV_i = \frac{\sqrt{V_{ID}}}{\bar{x}}$

i.e. dividing the square root of the among-individual variance by the intercept (i.e. mean trait value).

```
CVi <- sqrt(posterior_animal_id) / summary(m1)$coefficients[1]
quantile(CVi,prob=c(0.025, 0.5, 0.975))
```

```
##      2.5%       50%     97.5%
## 0.1238892 0.1422252 0.1621406
```

Now, we may be interested on which elephants are the more or the less mobile ones. Such an information can, for example, be used when managing problem individuals or when estimating an individual's space requirements. In the behavioral ecology (and animal personality) literature this is called the "behavioral type". Statistically the behavioral type is the best linear unbiased prediction (BLUP) of the random effect, i.e. the prediction for mean behavioral expression for each individual. We can use the merTools-package to obtain repeated samples from the posterior distribution of our model from which we can calculate the standard deviation for each level of the random effect.

```
randomSims <- REsim(m1, n.sims = 1000)
head(randomSims[randomSims$groupFctr=="animal_id",])
```

```
##    groupFctr    groupID        term      mean    median       sd
## 78 animal_id  elephant1 (Intercept)  2.361753  2.328272 1.221967
## 79 animal_id elephant10 (Intercept)  2.558298  2.600158 1.617226
## 80 animal_id elephant11 (Intercept) -1.666162 -1.646776 1.052494
## 81 animal_id elephant13 (Intercept)  5.223139  5.276265 1.007446
## 82 animal_id elephant15 (Intercept) -2.383780 -2.392727 1.062460
## 83 animal_id elephant16 (Intercept)  1.269859  1.329482 1.211758
```

```
# add the sex of the individual
randomSims <- merge(randomSims[randomSims$groupFctr=="animal_id",],
                  data[!duplicated(data$animal_id),c("animal_id","Sex")],
                    by.x = "groupID",by.y="animal_id")

# add identifier to color individuals uniquely
randomSims$ID <- ifelse(randomSims$groupID %in% c("elephant17", "elephant4", "elephant8",
                                                  "elephant36","elephant20"),
                   randomSims$groupID, "Other individuals")

# add population intercept and sex specific differences for easier
# interpretation of realized average daily movement distance
randomSims[randomSims$Sex == "F",]$mean <- randomSims[randomSims$Sex == "F",]$mean +
  fixef(m1)["(Intercept)"]
randomSims[randomSims$Sex == "M",]$mean <- randomSims[randomSims$Sex == "M",]$mean +
  (fixef(m1)["(Intercept)"] + fixef(m1)["SexM"])

# sort data
randomSims$groupID <- factor(randomSims$groupID,
                             levels = randomSims$groupID[order(randomSims$mean)])

ggplot()+
  geom_errorbar(data = randomSims,
```

```r
                 aes(x = groupID, ymin = mean-sd,
                     ymax = mean+sd, color = Sex))+
  geom_point(data = randomSims,
             aes(x = groupID, y = mean, fill = ID), shape = 21, size = 2)+
  theme_classic()+
  ylab("BT mean daily distance (km)")+
  theme(axis.text.x = element_text(angle = 45, hjust = 1),
        axis.title.x = element_blank())+
  annotate("text",x = 30, y = 12, label = "R = 0.22*", size = 4)+
    scale_fill_manual(values = c("#F8766D","#C77CFF","#7CAE00","#FFCC00", "#00BFC4","gray"))
```

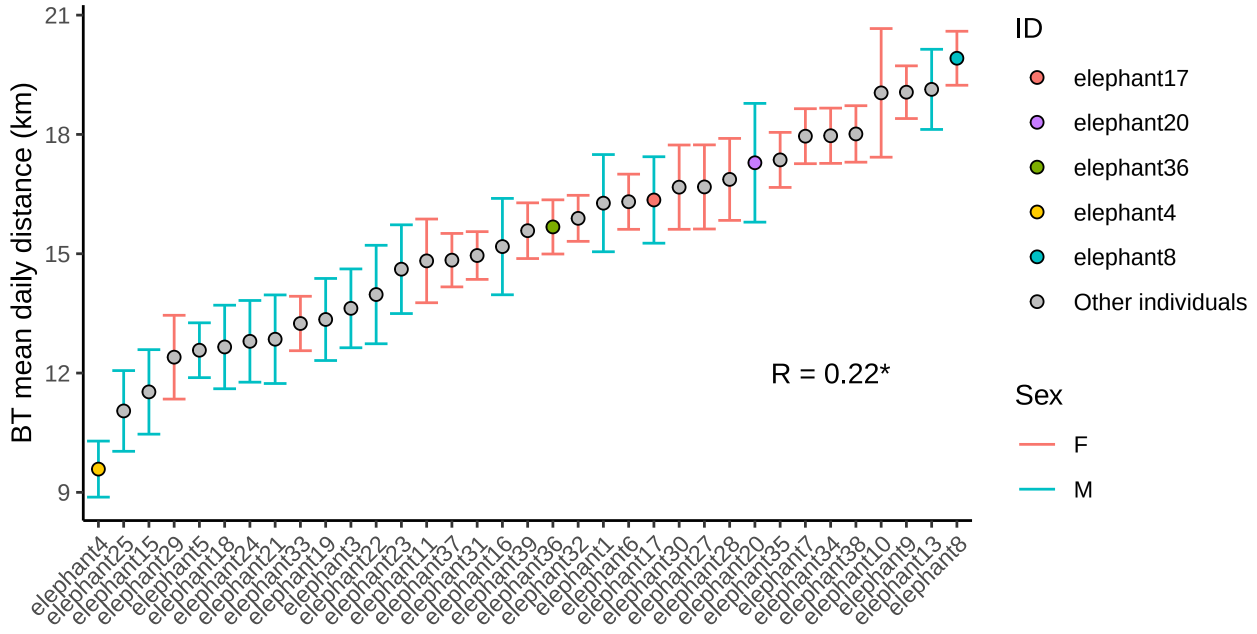

Figure 9: Behavioral type for daily movement distance.

In our example elephant4 moves over the shortest distances of all elephants (about 9.5 km, Fig 9). Elephant8 moves over the longest daily distances (about 20 km). Generally male elephants move over shorter daily distances than females, which was already demonstrated earlier. We can repeat the analysis for residence time (RT) and turn angle correlation (TAC).

Behavioral types and repeatability of residence time & turn angle correlation

```r
m2 <- lmer(meanRT ~ month + I(month^2)  + Sex + (1|animal_id)+ (1|year/month), data ,REML=T)

m3 <- lmer(tac ~ month + I(month^2)  + Sex + (1|animal_id)+ (1|year/month), data ,REML=T)
```

Repeatability (R) for mean residence time is estimated as 0.17 [0.13, 0.22] and for turn angle correlation as 0.31 [0.26, 0.36]

```r
quantile(posterior_animal_id.m2 /
        (posterior_animal_id.m2 + posterior_yearmonth.m2 + posterior_year.m2 + posterior_residual.m2),
         prob=c(0.025, 0.5, 0.975))
```

```
##      2.5%       50%     97.5%
```

```
## 0.1297046 0.1676830 0.2165311
```

```r
quantile(posterior_animal_id.m3 /
        (posterior_animal_id.m3 + posterior_yearmonth.m3 + posterior_year.m3 + posterior_residual.m3),
         prob=c(0.025, 0.5, 0.975))
```

```
##      2.5%       50%     97.5%
## 0.2612445 0.3024222 0.3526933
```

Coefficient of variation for between individual variance (CV_i) was 0.14 for movement distance, and is 0.16 for residence time and 0.08 for turn angle correlation. This indicates that within-individual (as opposed to among-individual) variation may be much larger in turn angle correlation than in movement distance and residence time.

```r
quantile(sqrt(posterior_animal_id.m2) / summary(m2)$coefficients[1],prob=c(0.025, 0.5, 0.975))
```

```
##      2.5%       50%     97.5%
## 0.1403400 0.1628662 0.1914706
```

```r
quantile(sqrt(posterior_animal_id.m3) / summary(m3)$coefficients[1],prob=c(0.025, 0.5, 0.975))
```

```
##       2.5%        50%      97.5%
## 0.07704583 0.08490835 0.09507379
```

Elephants range in their average behavioral expression for residence time from 2.3 - 3.5 hours (Fig 10) and in their average behavioral expression for turn angle correlation from 0.6 - 0.88 (Fig 11).

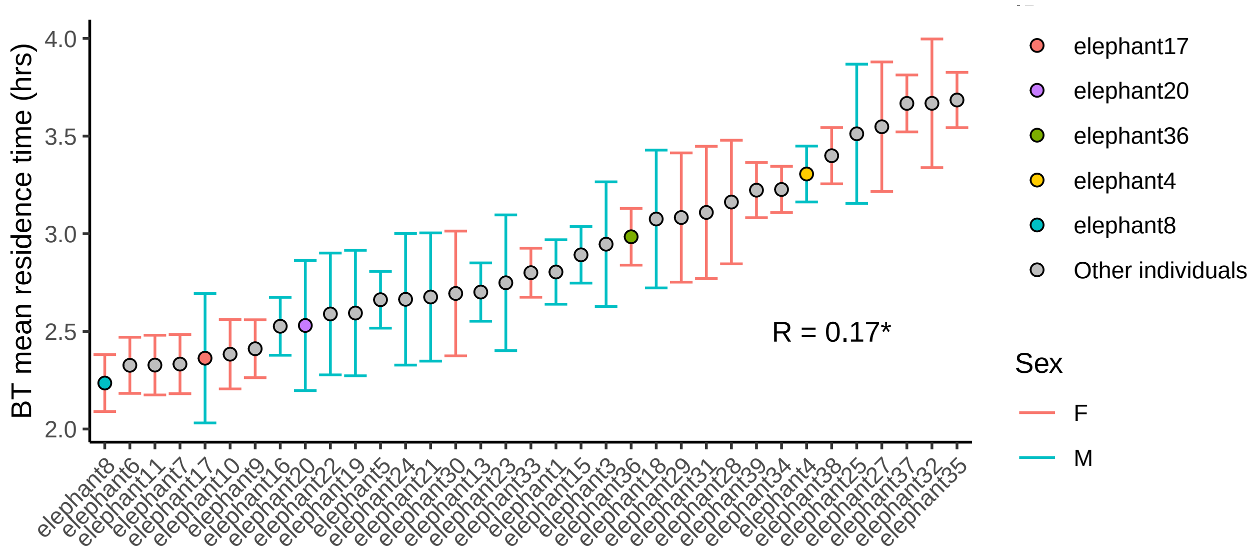

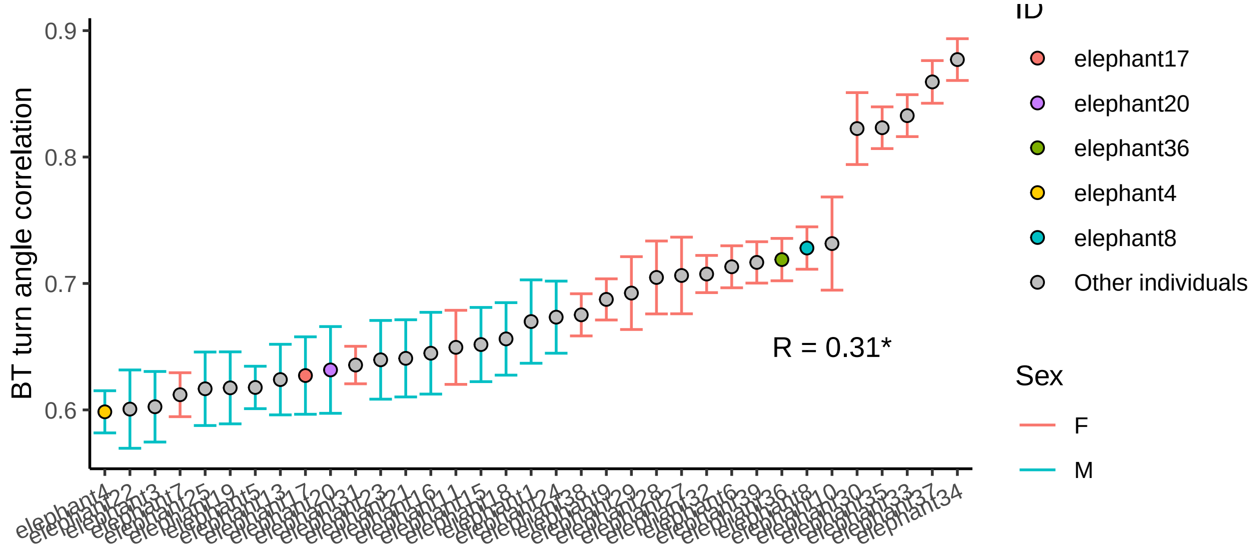

Bayesian Approach

Alternatively to a frequentist approach where we use lme4 to fit a mixed model we can also use a Bayesian approach. We expect more or less similar results between the two approaches. A practical advantage of a Bayesian modelling approach is that the model gives a posterior distribution for each estimated parameter and hence inherently reflects uncertainty. We here use the brms package for model fitting. We use an uninformative prior, the default in brms. As mentioned previously we will not go into detail how to select and fit priors, but see Hadfield (2010) for more detail on prior selection.
Hadfield (2010) "MCMC methods for multi-response generalized linear mixed models: the MCMCglmm R package."

The second function below adds the model selection criterion "WAIC" to the model output which we will use later on.

```
my.cores <- detectCores()
m1_brm <- brm(meanDailyDisplacement ~ month + I(month^2) + Sex +
                                (1 | animal_id) + (1 | year/month),
              data = data,
              warmup = 500,
              iter = 3000,
              thin=2,
              chains = 2,
              inits  = "random",
              cores  = my.cores,
              seed = 12345)

m1_brm <- add_criterion(m1_brm, "waic")
```

```
m1_brm <- readRDS("m1_brm.rds")
summary(m1_brm)
```

```
##  Family: gaussian
##   Links: mu = identity; sigma = identity
## Formula: meanDailyDisplacement ~ month + I(month^2) + Sex + (1 | animal_id) + (1 | year/month)
##    Data: data (Number of observations: 3766)
## Samples: 2 chains, each with iter = 3000; warmup = 500; thin = 2;
##          total post-warmup samples = 2500
##
```

```
## Group-Level Effects:
## ~animal_id (Number of levels: 35)
##               Estimate Est.Error l-95% CI u-95% CI Rhat Bulk_ESS Tail_ESS
## sd(Intercept)     2.31      0.31     1.80     2.99 1.00      851     1577
##
## ~year (Number of levels: 8)
##               Estimate Est.Error l-95% CI u-95% CI Rhat Bulk_ESS Tail_ESS
## sd(Intercept)     0.60      0.37     0.07     1.46 1.00      935     1058
##
## ~year:month (Number of levels: 77)
##               Estimate Est.Error l-95% CI u-95% CI Rhat Bulk_ESS Tail_ESS
## sd(Intercept)     1.06      0.14     0.81     1.35 1.00     1635     2016
##
## Population-Level Effects:
##           Estimate Est.Error l-95% CI u-95% CI Rhat Bulk_ESS Tail_ESS
## Intercept    16.48      0.78    14.97    18.03 1.00      905     1285
## month        -0.96      0.18    -1.32    -0.62 1.00     1502     1781
## ImonthE2      0.07      0.01     0.04     0.09 1.00     1499     2117
## SexM         -2.58      0.80    -4.17    -1.01 1.00      573      883
##
## Family Specific Parameters:
##       Estimate Est.Error l-95% CI u-95% CI Rhat Bulk_ESS Tail_ESS
## sigma     4.29      0.05     4.19     4.39 1.00     1859     1436
##
## Samples were drawn using sampling(NUTS). For each parameter, Bulk_ESS
## and Tail_ESS are effective sample size measures, and Rhat is the potential
## scale reduction factor on split chains (at convergence, Rhat = 1).
```

We should make it a habit to inspect a) the effective sample size and b) the Rhat parameter to make sure that our model converged. In a nutshell, we want "Eff.Sample" close to the expected sample size for all parameters. The expected sample size is the number of iterations minus the number of iterations which we discard as warm-up and divided by the thinning interval and multiplied by the number of chains defined in the model (2 chains in our case), i.e. ((3000 - 500) / 2) * 2 = 2500 Rhat on the other hand should be 1.

Similar to the frequentist approach, we can calculate repeatability by dividing the posterior distribution for the variance explained by the random intercept animal_id by the total variance. *Importantly* - in brms variance parameters are given in standard deviations and need to be squared to calculate the variance!

We can either calculate repeatability by hand:

```
(2.31)^2 / ((2.31)^2 + (0.6)^2 + (1.06)^2 + (4.29)^2)
```

```
## [1] 0.2115502
```

Or (better) take the mean and credible interval of the posterior distribution.

```
colnames(posterior_samples(m1_brm))[1:8]
```

```
## [1] "b_Intercept"                "b_month"
## [3] "b_ImonthE2"                 "b_SexM"
## [5] "sd_animal_id__Intercept"  "sd_year__Intercept"
## [7] "sd_year:month__Intercept" "sigma"
```

```
var.animal_id <- posterior_samples(m1_brm)$"sd_animal_id__Intercept"^2
var.year <- posterior_samples(m1_brm)$"sd_year__Intercept"^2
var.year.month <- posterior_samples(m1_brm)$"sd_year:month__Intercept"^2
var.res <- posterior_samples(m1_brm)$"sigma"^2

RDist <- var.animal_id / (var.animal_id + var.year.month + var.year + var.res)
mean(RDist);HPDinterval(as.mcmc(RDist),0.95)
```

```
## [1] 0.2104436

##          lower     upper
## var1 0.1323902 0.3002388
## attr(,"Probability")
## [1] 0.95
```

The mean and credible interval for this posterior distribution is 0.21 [0.13, 0.3], so similar but slightly higher than our result in the frequentist approach. It means that after controlling for the fixed effects of month and Sex - 21% of the remaining variance can be explained by individual differences in behavioral expression among elephants.

```
RYear <- var.year / (var.animal_id + var.year.month + var.year + var.res)
mean(RYear)
```

```
## [1] 0.01901271
```

```
RYearMonth <- var.year.month / (var.animal_id + var.year.month + var.year + var.res)
mean(RYearMonth)
```

```
## [1] 0.04519953
```

```
RRes <- var.res / (var.animal_id + var.year.month + var.year + var.res)
mean(RRes)
```

```
## [1] 0.7253441
```

Similar, and as explained in the frequentist section, we can calculate CV_i as:

```
CVi <- sqrt(var.animal_id) / mean(data$meanDailyDisplacement)
mean(CVi);HPDinterval(as.mcmc(CVi),0.95)
```

```
## [1] 0.177232

##          lower     upper
## var1 0.1337719 0.2237333
## attr(,"Probability")
## [1] 0.95
```

In summary, 21% of the variance can be attributed to individual differences in average movement, only 2% of the variance can be explained by differences between years in average movement, 5% due to consistent month effects and 72% of the variance is unexplained residual variance, i.e. within-individual, within-year, within-month variation and measurement error.

Be aware - Bayesian 95% credible intervals that do not cross zero are commonly used to indicate statistical significance. This is, however, not true for variance components which are by definition always positive. Low credible interval bounds close to zero therefore indicate a low confidence into a statistical significance of the repeatability estimate which is for example the case for the year and month in year random effects. One way to get more insight about this issue is to plot the distribution for the focal random effect and see whether the distribution hits the "zero wall".

Next we can go ahead and, similar to the frequentist approach, plot individual level distributions for visual inspection of individual differences in behavioral types for movement distance.

We add the population level intercept (i.e. mean value) and coefficient for it's sex (male or female) to each elephant's posterior distribution to make behavioral types more interpretable. This way, the individual level posterior distributions represent the true trait values.

```
posteriorBT <- posterior_samples(m1_brm)[,9:43] %>%
  gather(animal_id, value,
         "r_animal_id[elephant1,Intercept]" : "r_animal_id[elephant9,Intercept]")%>%
  separate(animal_id,
           c(NA,NA,NA,"animal_id",NA),
```

```
          sep = "([\\_\\[\\,])", fill = "right") %>%
  left_join(select(data[!duplicated(data$animal_id),], animal_id, Sex))

posteriorBT[posteriorBT$Sex == "F",]$value <-
  posteriorBT[posteriorBT$Sex == "F",]$value + fixef(m1_brm, pars = "Intercept")[1]

posteriorBT[posteriorBT$Sex == "M",]$value <-
  posteriorBT[posteriorBT$Sex == "M",]$value + fixef(m1_brm, pars = "Intercept")[1] +
  fixef(m1_brm, pars = "SexM")[1]

posteriorBT$col <- ifelse(posteriorBT$animal_id %in%
                            c("elephant17", "elephant4", "elephant8",
                              "elephant36","elephant20"),
                          posteriorBT$animal_id, "Other individuals")

posteriorBT <- posteriorBT %>%
  dplyr::group_by(animal_id) %>%
  dplyr::mutate(meanBT = mean(value))%>%
  dplyr::ungroup()

BT <- ggplot()+
  ggridges::geom_density_ridges(data = posteriorBT,
                                aes(x = value,
                                    y = reorder(as.factor(animal_id), meanBT),
                                    height = ..density..,
                                    fill = col,scale = 3), alpha = 0.6)+
            geom_point(data = posteriorBT[!duplicated(posteriorBT$animal_id),],
             aes(x = meanBT,
                 y = as.factor(animal_id),
                 col = Sex),
             size = 1)+
            labs(y = "",
                 x = "BT mean daily distance (km)",
                 fill = "ID")+
            theme_classic()+
    scale_fill_manual(values = c("#F8766D","#C77CFF","#7CAE00",
                                 "#FFCC00","#00BFC4","gray"))
```

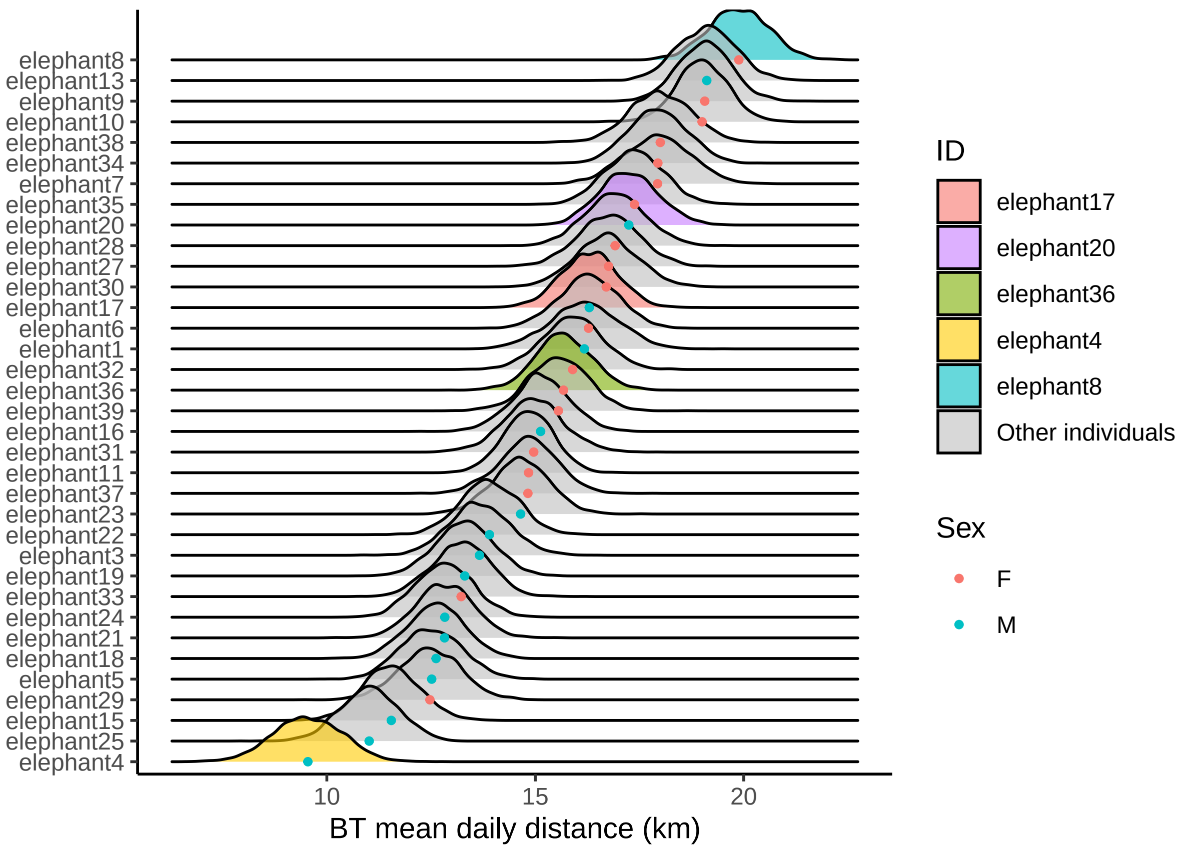

Figure 10: Behavioral type for movement distance.

Variation in behavioral plasticity: Random regression models

In general any environmental or experimental gradient along which we expect behavioral plasticity and along which we have measured an individual multiple times can be used to analyze individual variation in plasticity.

Frequentist approach

Do elephants differ in how they shift movement between dry and wet seasons?: individual variation in plasticity

We fit *random intercepts* for animal_id and individual *random slopes* for the environmental gradient (i.e. interaction term between animal_id and environmental variable as a random effect) to test if individuals differ in how they shift their behavior across an environmental gradient. As we saw earlier, elephants shift their behavior in a non-linear fashion over months. We will simplify the here presented example and only test how individuals shift their daily movement distances between the hot-wet season in Etosha (January - March) to the cold-dry season (June - August). To facilitate model convergence we need to check that all individuals were monitored in both seasons.

```
RN <- data[data$month %in% c(1,2,3,6,7,8),]
RN$season <- ifelse(RN$month %in% c(1,2,3), "wet","dry")
table(RN$season,RN$animal_id)
```

```
##
##        elephant1 elephant10 elephant11 elephant13 elephant15 elephant16
##   dry         13         60         56         33         33         26
```

```
##   wet        12          56          66          27          27          27
##
##       elephant17 elephant18 elephant19 elephant20 elephant21 elephant22
##   dry          29          26          26          27          26          28
##   wet          26          27          27          27          27          27
##
##       elephant23 elephant24 elephant25 elephant27 elephant28 elephant29
##   dry          29          28          29          22          23          22
##   wet          27          27          27          25          25          25
##
##       elephant3 elephant30 elephant31 elephant32 elephant33 elephant34
##   dry        20          23          23          23          20          20
##   wet        27          25          24          23          24          20
##
##       elephant35 elephant36 elephant37 elephant38 elephant39 elephant4
##   dry          21          21          19          12          13          11
##   wet          24          25          23          24          32           9
##
##       elephant5 elephant6 elephant7 elephant8 elephant9
##   dry        26        26        13        25        49
##   wet        26        27        16        27        56
```

We start with comparing two models - one with a random intercept for animal_id and one with a random intercept for animal_id AND a random slope over season for each animal (i.e. interaction between the season and animal_id as a random effect).

```
ri <- lmer(meanDailyDisplacement ~ season + Sex +
             (1|animal_id), RN)
ris <- lmer(meanDailyDisplacement ~ season + Sex +
              (season|animal_id), RN)
AIC(ri,ris)
```

```
##     df      AIC
## ri   5 10945.45
## ris  7 10719.09
```

```
summary(ris)
```

```
## Linear mixed model fit by REML ['lmerMod']
## Formula: meanDailyDisplacement ~ season + Sex + (season | animal_id)
##    Data: RN
##
## REML criterion at convergence: 10705.1
##
## Scaled residuals:
##     Min      1Q  Median      3Q     Max
## -3.3267 -0.5502 -0.1316  0.4237  6.6878
##
## Random effects:
##  Groups    Name        Variance Std.Dev. Corr
##  animal_id (Intercept)  7.975   2.824
##            seasonwet   13.233   3.638    -0.47
##  Residual              16.511   4.063
## Number of obs: 1865, groups:  animal_id, 35
##
## Fixed effects:
```

```
##              Estimate Std. Error t value
## (Intercept)  12.7130     0.6386  19.909
## seasonwet     1.4736     0.6465   2.279
## SexM         -1.5336     0.8698  -1.763
##
## Correlation of Fixed Effects:
##           (Intr) sesnwt
## seasonwet -0.389
## SexM      -0.625  0.004
```

The more complex model including the interaction between the season and individual identity fits better. It seems that elephants shift their behavior differently between the two seasons. The negative correlation of -0.47 between the random intercept and slope indicates that those individuals which had a longer daily movement distance during the dry season increased their movement less strong during the wet season as compared to individuals with a shorter movement distance during the dry season. To make this relationship clearer, lets plot our model predictions and raw data:

```
RI<-augment(ri) %>%
  dplyr::select(meanDailyDisplacement, season , animal_id, .fitted)

RIS<-augment(ris) %>%
  dplyr::select(meanDailyDisplacement, season, animal_id, .fitted)

RI$.fittedRIS<-RIS$.fitted

df <- RI %>%
  dplyr::group_by(animal_id, season) %>%
  dplyr::summarise(RI = mean(.fitted),
           RIS = mean(.fittedRIS))%>%
  gather(type, Value, `RI`:`RIS`)
df$animal_id <- as.character(df$animal_id)
df$ID <- ifelse(df$animal_id %in%
                            c("elephant17", "elephant4", "elephant8",
                              "elephant36","elephant20"),
                          df$animal_id, "Other Individuals")

plot_ri <- ggplot(df[df$type == "RI",], aes(x = season, y = Value,
                 group = animal_id,color = ID)) +
  geom_line() +
  theme_classic() +
  labs(y="", x="")+
  ggtitle("Random Intercept")+
  ylim(4,23)+
  scale_color_manual(values = c("#F8766D","#C77CFF","#7CAE00","#FFCC00",
                               "#00BFC4","gray"))+
  guides(color = guide_legend(nrow = 2, byrow = TRUE))

plot_ris <- ggplot(df[df$type == "RIS",], aes(x = season, y = Value,
                                     group = animal_id,color = ID)) +
  geom_line() +
  theme_classic()+
  labs(y="", x="")+
  ggtitle("Random Intercept\n and Slope")+
  ylim(4,23)+
  scale_color_manual(values = c("#F8766D","#C77CFF","#7CAE00","#FFCC00",
```

```
                                   "#00BFC4","gray"))
mylegend<-g_legend(plot_ri)

grid.arrange(
  arrangeGrob(plot_ri+ theme(legend.position="none"),
              plot_ris+ theme(legend.position="none"),
              ncol=2),mylegend,heights=c(10, 2))
```

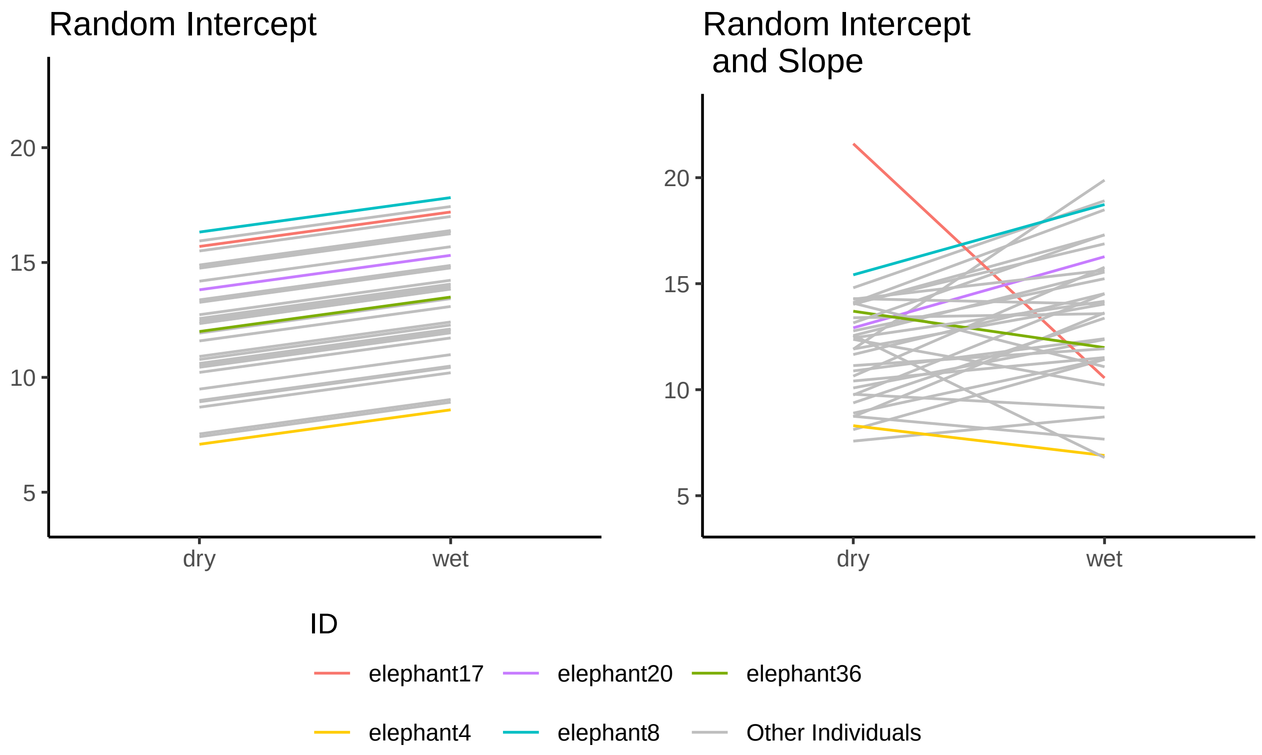

Figure 11: Individual shifts of movement between the dry and wet season: (left) Prediction lines assuming elephants adjust behavior equally between the two seasons (random intercept), (right) Prediction lines assuming elephants differ in the extent to which they shift movement distance between the two seaons (random intercept and slope).

As we can see in the observed data, not all individuals increase their daily movement distance in the wet season compared to their daily movement distance during the dry season in a similar way (Fig 12 left). In fact there are few individuals that reduce movements and few that only slightly increase movements. The random regression model accounts for these differences in behavioral plasticity of movement towards seasonal changes(Fig 12 right).

Bayesian approach

Do elephants differ in how they shift movement over the course of the year?: individual variation in plasticity

Now given that elephants apparently differ in how they adjust their behavior between dry and wet season, we may want to revise our original model from section 1 and test whether a nonlinear random slope over month improves model fit.

```r
m2_brm <- brm(meanDailyDisplacement ~ poly(month,2) + Sex +
                                (poly(month,2)| animal_id) + (1 | year/month),
              data  = data,
              warmup = 500,
              iter = 3000,
              thin=2,
              chains = 2,
              inits  = "random",
              seed = 12345,
              cores  = my.cores)
m2_brm <- add_criterion(m2_brm, "waic")
```

```r
m2_brm <- readRDS("m2_brm.rds")
comp <- loo_compare(m1_brm, m2_brm, criterion = "waic")
print(comp, simplify = FALSE, digits = 3)
```

```
##         elpd_diff  se_diff    elpd_waic  se_elpd_waic p_waic     se_p_waic
## m2_brm      0.000      0.000 -10612.452     77.331      147.647      6.801
## m1_brm   -262.066     25.733 -10874.518     80.454       87.451      4.395
##         waic       se_waic
## m2_brm  21224.904    154.662
## m1_brm  21749.036    160.908
```

```r
deltaWAIC <- 21749.036 - 21224.904
deltaWAIC
```

```
## [1] 524.132
```

We use the Widely Applicable Information Criterion (WAIC) for model comparison. Similar to AIC, a smaller WAIC indicates a better predictive model performance. If the difference in WAIC between the two models (deltaWAIC) is larger than 7 we can infer that the more complicated model indeed provides a better fit. In our case we get a deltaAIC of 524, the model comparison between the intercept and slope and the intercept only model (section 1), therefore cleary indicates that the more complicated model produces a better model fit. This means that elephants shift their movement behavior differently over the year.

```r
summary(m2_brm)
```

```
##  Family: gaussian
##   Links: mu = identity; sigma = identity
## Formula: meanDailyDisplacement ~ poly(month, 2) + Sex + (poly(month, 2) | animal_id) + (1 | year/month)
##    Data: data (Number of observations: 3766)
## Samples: 2 chains, each with iter = 3000; warmup = 500; thin = 2;
##          total post-warmup samples = 2500
##
## Group-Level Effects:
## ~animal_id (Number of levels: 35)
##                              Estimate Est.Error l-95% CI u-95% CI Rhat Bulk_ESS Tail_ESS
## sd(Intercept)                    2.39      0.32     1.86     3.09 1.01      686     1238
## sd(polymonth21)                 67.08      9.48    51.48    87.10 1.00     1141      503
## sd(polymonth22)                 79.62     11.21    61.62   105.03 1.00      745      358
## cor(Intercept,polymonth21)      -0.02      0.18    -0.37     0.33 1.00      700     1186
## cor(Intercept,polymonth22)       0.27      0.17    -0.07     0.59 1.00      679      387
## cor(polymonth21,polymonth22)    -0.06      0.17    -0.39     0.27 1.00      850     1569
##
## ~year (Number of levels: 8)
##               Estimate Est.Error l-95% CI u-95% CI Rhat Bulk_ESS Tail_ESS
## sd(Intercept)     0.69      0.50     0.13     1.62 1.00      707      542
##
```

```
## ~year:month (Number of levels: 77)
##               Estimate Est.Error l-95% CI u-95% CI Rhat Bulk_ESS Tail_ESS
## sd(Intercept)     1.01      0.14     0.77     1.29 1.00     1076     1385
##
## Population-Level Effects:
##             Estimate Est.Error l-95% CI u-95% CI Rhat Bulk_ESS Tail_ESS
## Intercept      13.78      0.63    12.56    14.94 1.01      336      406
## polymonth21   -25.01     13.95   -51.13     2.02 1.01      880     1532
## polymonth22    35.56     16.40     3.76    68.34 1.00      823     1206
## SexM           -2.12      0.88    -3.78    -0.33 1.01      297      600
##
## Family Specific Parameters:
##       Estimate Est.Error l-95% CI u-95% CI Rhat Bulk_ESS Tail_ESS
## sigma     3.97      0.05     3.88     4.06 1.00     2374     1941
##
## Samples were drawn using sampling(NUTS). For each parameter, Bulk_ESS
## and Tail_ESS are effective sample size measures, and Rhat is the potential
## scale reduction factor on split chains (at convergence, Rhat = 1).
```

As we can see in the summary output - both polynomial terms of the random slope are "significant" and the 95% credible intervals do not include 0 (poly1 = 66.73 [50.83;87.86], poly2 = 79.26 [61.70;102.76]). However, different than in the frequentist example, the correlation between random intercept and slope is not supported. In the previous example, elephants that moved less during the dry season increased their movement more strongly during the wet season as compared to elephants that already moved more during the dry season (Fig 11). In the current example we find that elephants differ in how they shift their movement distance over the course of the year (Fig 12) but the rate of shifting is unrelated to their behavioral type.

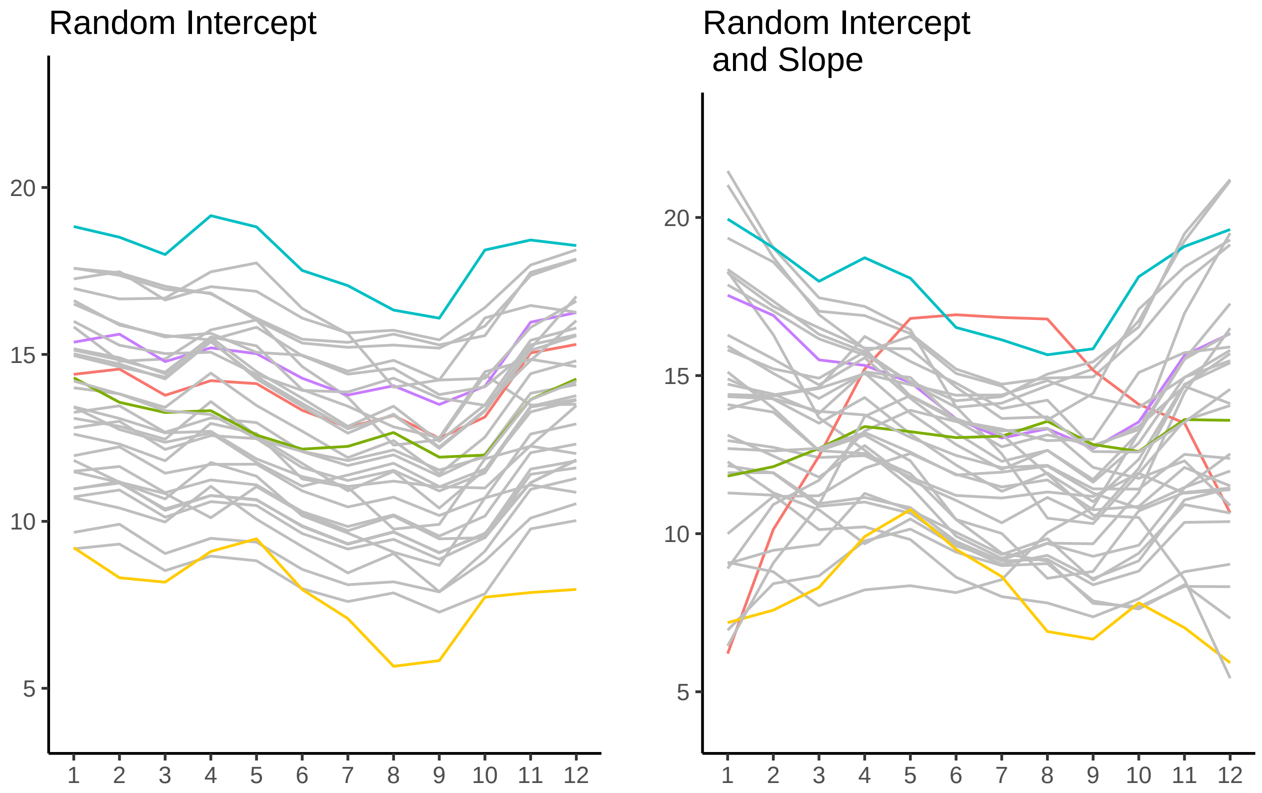

Figure 12: Individual shifts of daily movement distance over month of the year: (left) In a random intercept only model, elephants are not expected to differ in how they adjust movement over study month. (right) When controlling for individual variation in shifts of movement distance over the course of the year with a random regression model, we find that elephants indeed differ in how they shift movement distance over study months.

Variation in behavioral predictability: Double hierarchical mixed effects models

In addition to temporal plasticity, i.e. the extent to which elephants change their behavior over study months, we may also suspect that elephants additionally differ in their predictability of behavior. Perhaps some individuals are behaviorally more flexible while others are behaviorally more rigid. We can measure individual variation in predictability by estimating variation in residual intra-individual variation (rIIV), i.e. the spread of residuals around an individuals reaction norm. There have been several statistical measures proposed to quantify this type of variation (see Cleasby et al. 2015). We would like to highlight that double-hierarchical GLM's (DHGLM) are the statistically soundest approach to estimate among-individual variation in intra-individual variance:

Cleasby I., Nagakawa S., Schielzeth H. (2015) Quantifying the predictability of behavior: statistical approaches for the study of between-individual variation in the within-individual variance. Methods in Ecology and Evolution. doi: 10.1111/2041-210X.12281

We here present a relatively simple way to fit DHLGM's to our elephant data using the brms-package. Additionally to the variance structure on the "mean" part of the response variable, brms allows us to also impose a variance structure onto the residual part of the variance (i.e. "sigma"). For example - we can use DHGLM's to partition the residual variance per individual. Individuals with a high residual variance are accordingly more unpredictable than individuals with a low residual variance.

```r
double_model = bf(meanDailyDisplacement ~
                    poly(month,2) + Sex + (poly(month,2)|animal_id),
                  sigma ~ (1|animal_id))

m3_brm <- brm(double_model,
              data   = data,
              warmup = 500,iter = 3000, thin=2,
              chains = 2, inits  = "random",
              seed = 12345,
              cores  = my.cores)
```

```r
m3_brm <- readRDS("m3_brm.rds")
summary(m3_brm)
```

```
##  Family: gaussian
##   Links: mu = identity; sigma = log
## Formula: meanDailyDisplacement ~ poly(month, 2) + Sex + (poly(month, 2) | animal_id)
##          sigma ~ (1 | animal_id)
##    Data: data (Number of observations: 3766)
## Samples: 2 chains, each with iter = 3000; warmup = 500; thin = 2;
##          total post-warmup samples = 2500
##
## Group-Level Effects:
## ~animal_id (Number of levels: 35)
##                              Estimate Est.Error l-95% CI u-95% CI Rhat Bulk_ESS Tail_ESS
## sd(Intercept)                    2.44      0.32     1.93     3.14 1.00      741     1219
## sd(polymonth21)                 66.82      9.68    49.85    88.83 1.00     1376     1535
## sd(polymonth22)                 71.65     10.11    54.06    93.76 1.00     1275     1946
## sd(sigma_Intercept)              0.27      0.04     0.21     0.35 1.00      835     1336
## cor(Intercept,polymonth21)      -0.02      0.18    -0.37     0.32 1.00      775     1176
## cor(Intercept,polymonth22)       0.28      0.17    -0.08     0.58 1.00      893     1407
## cor(polymonth21,polymonth22)     0.01      0.18    -0.34     0.36 1.00      878     1532
##
## Population-Level Effects:
##                 Estimate Est.Error l-95% CI u-95% CI Rhat Bulk_ESS Tail_ESS
## Intercept          13.76      0.57    12.61    14.87 1.01      332      457
## sigma_Intercept     1.34      0.05     1.24     1.43 1.00      484      834
## polymonth21       -17.31     11.67   -40.97     5.11 1.01      773     1447
## polymonth22        38.72     13.46    11.45    64.68 1.00      720     1421
## SexM               -1.99      0.85    -3.68    -0.30 1.01      386      660
##
## Samples were drawn using sampling(NUTS). For each parameter, Bulk_ESS
## and Tail_ESS are effective sample size measures, and Rhat is the potential
## scale reduction factor on split chains (at convergence, Rhat = 1).
```

The model output looks very similar to the output in the previous section on random regression models, except that we find an additional term under "Population-Level Effects": "sigma_Intercept" and another additional two terms under "Group-Level Effects": "sd(sigma_Intercept)". Similar to a population intercept, "sigma_Intercept" is the population level estimate of the residual variance. "sd(sigma_Intercept)" on the other hand is the estimate for individual differences in residual variance.

"sd(sigma_Intercept)" indicates whether there is support for individual variation in residual variance. In this case, we find among-individual variation in predictability $\omega_{ID} = 0.27$ [0.22 - 0.35].

Since sigma components are given in standard deviations on the log-scale we need to first exponentiate the estimate and then square it to calculate the variance.

```r
head(get_variables(m3_brm),10)
```

```
##  [1] "b_Intercept"
##  [2] "b_sigma_Intercept"
##  [3] "b_polymonth21"
##  [4] "b_polymonth22"
##  [5] "b_SexM"
##  [6] "sd_animal_id__Intercept"
##  [7] "sd_animal_id__polymonth21"
##  [8] "sd_animal_id__polymonth22"
##  [9] "sd_animal_id__sigma_Intercept"
## [10] "cor_animal_id__Intercept__polymonth21"
```

```r
var.res <- exp(posterior_samples(m3_brm)$"sd_animal_id__sigma_Intercept")^2
mean(var.res);HPDinterval(as.mcmc(var.res),0.95)
```

```
## [1] 1.710455

##         lower    upper
## var1 1.499812 1.975022
## attr(,"Probability")
## [1] 0.95
```

We can also compute the "coefficient of variation in predictability" (CV_P) a standardized population-level measure of the degree of variation in predictability among all our study individuals. CV_P can be used to compare the degree of among-individual variation in predictability across studies and traits and we therefore recommend authors always to report it.

$CV_P = \sqrt{\exp(\omega_{ID}^2) - 1}$

$CV_P = 0.27$ [0.2, 0.35]

```r
log.norm.res <- exp(posterior_samples(m3_brm)$"sd_animal_id__sigma_Intercept"^2)
CVP <- sqrt(log.norm.res - 1)
mean(CVP);HPDinterval(as.mcmc(CVP),0.95)
```

```
## [1] 0.272176

##          lower     upper
## var1 0.2047689 0.3503828
## attr(,"Probability")
## [1] 0.95
```

Finally, similar to BLUPs in the first section we can plot the posterior distribution of each individual's predicted standard deviation (i.e. rIIV). Individuals with higher rIIV are less predictable than individuals with lower rIIV (Fig).

```r
posteriorIIV <- posterior_samples(m3_brm)[,118:152] %>%
  gather(animal_id, value,
         "r_animal_id__sigma[elephant1,Intercept]" : "r_animal_id__sigma[elephant9,Intercept]")%>%
  separate(animal_id, c(NA,NA,NA,NA,NA,"animal_id",NA),
           sep = "([\\__\\[\\,])", fill = "right") %>%
  dplyr::left_join(dplyr::select(data, animal_id, Sex))

# add population level mean residual variance
posteriorIIV$value <-
  posteriorIIV$value +
  fixef(m3_brm, pars = "sigma_Intercept")[1]
```

```r
posteriorIIV <- posteriorIIV %>%
  dplyr::group_by(animal_id) %>%
  dplyr::mutate(meanIIV = mean(value))%>%
  dplyr::ungroup()

posteriorIIV$ID <- ifelse(posteriorIIV$animal_id
                           %in% c("elephant17", "elephant4", "elephant8",
                                  "elephant36","elephant20"),
                           posteriorIIV$animal_id, "Other Individuals")

IIV <- ggplot()+
  ggridges::geom_density_ridges(data = posteriorIIV,
                                aes(x = value,
                                    y = reorder(as.factor(animal_id), meanIIV),
                                    height = ..density..,
                                    fill = ID,scale = 3), alpha = 0.6)+
            geom_point(data = posteriorIIV[!duplicated(posteriorIIV$animal_id),],
             aes(x = meanIIV,
                 y = as.factor(animal_id),
                 col = Sex),
             size = 1)+
             labs(y = "",
                 x = "Intraindividual variance (rIIV)",
                 fill = "ID")+
            scale_fill_manual(values = c("#F8766D","#C77CFF","#7CAE00","#FFCC00","#00BFC4","gray"))+
            theme_classic()
```

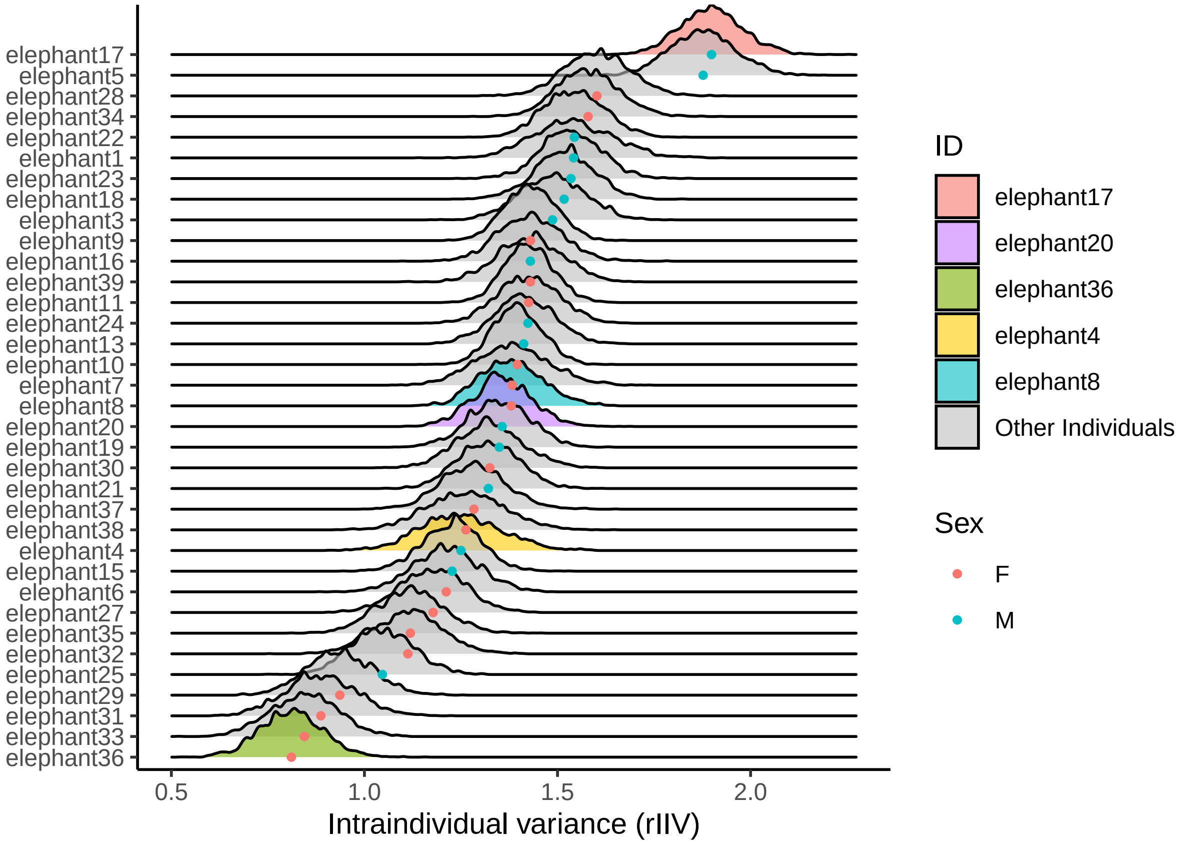

Figure 13: Individual variation in rIIV (on the log scale). Individuals with higher rIIV are less predictable than individuals with lower rIIV

Importantly, in order to assess whether individuals differ in variance the residual part of the model is calculated on the log scale! In order to interpret rIIV in biological terms we backtransform rIIV by taking it's original scale by taking its exponent.

```
posteriorIIV$value.exp <-
  exp(posteriorIIV$value)

posteriorIIV <- posteriorIIV %>%
  dplyr::group_by(animal_id) %>%
  dplyr::mutate(meanIIV.exp = mean(value.exp))%>%
  dplyr::ungroup()

expIIV <- ggplot()+
  ggridges::geom_density_ridges(data = posteriorIIV,
                                aes(x = value.exp,
                                    y = reorder(as.factor(animal_id), meanIIV.exp),
                                    height = ..density..,
                                    fill = ID,scale = 3), alpha = 0.6)+
            geom_point(data = posteriorIIV[!duplicated(posteriorIIV$animal_id),],
             aes(x = meanIIV.exp,
                 y = as.factor(animal_id),
                 col = Sex),
             size = 1)+
             labs(y = "",
```

```
            x = "Intraindividual variance (km)",
            fill = "ID")+
        scale_fill_manual(values = c("#F8766D","#C77CFF","#7CAE00","#FFCC00","#00BFC4","gray"))+
        theme_classic()
```

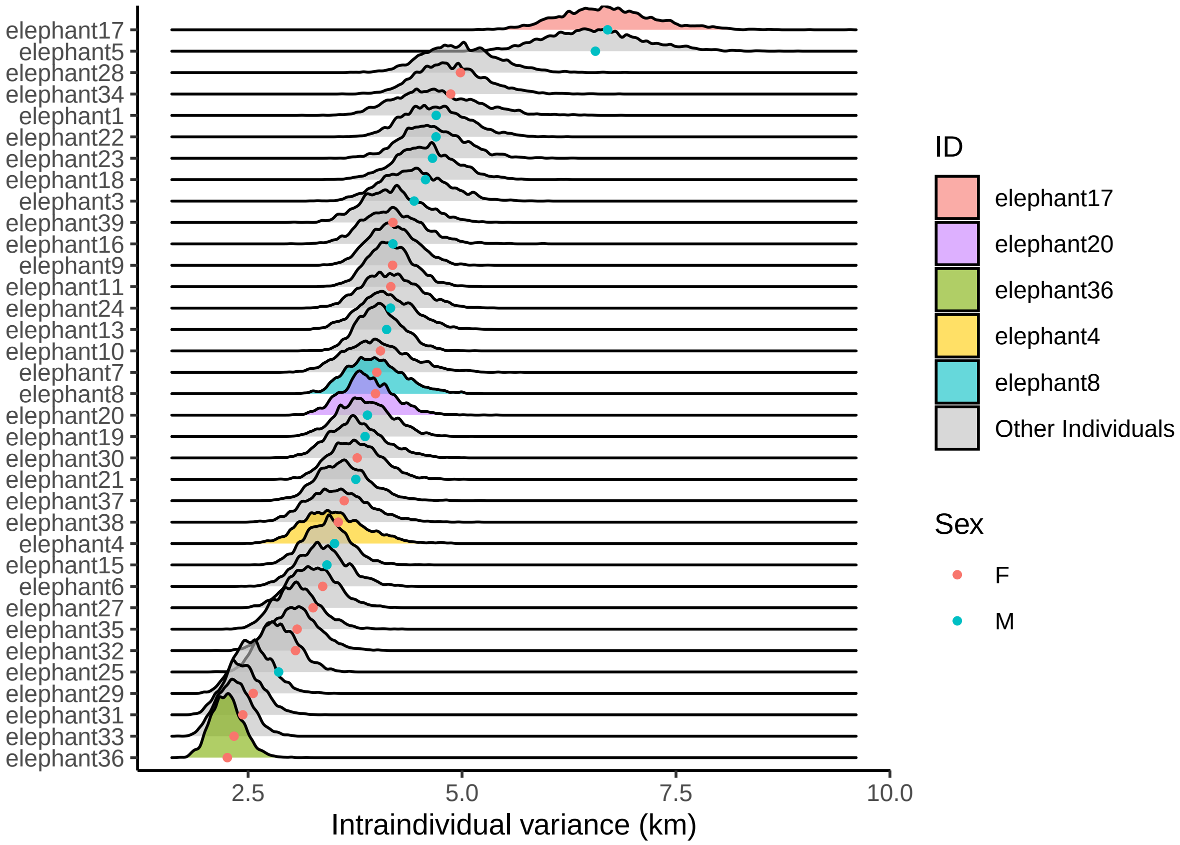

Figure 14: Backtransformed rIIV: The most predictable individual (elephant 36) has an average residual variance of 2.25km around its behavioral mean, whereas the least predictable individual (elephant 17) has an average residual variance of 6.7km

As we saw from the model estimate - individual elephants indeed differ in how predictable they are in their daily movement distance from ones that are highly predictable and consistent, characterized by a low intra-individual variance, to ones with high intra-individual variance and hence lower predictability.

It seems that males may be less predictable in their behavior compared to females. One reason for this could be because adult males are mostly solitary or in small bull groups and only join the female herd in search of a mate. Females on the other hand stay in larger herds of related females.

Specifically, elephant17 has the highest rIIV and is hence least predictable by our model, whereas elephant36 is most predictable, which becomes apparent when plotting the observed movement distance and the model predicted movement distance for the two individuals.

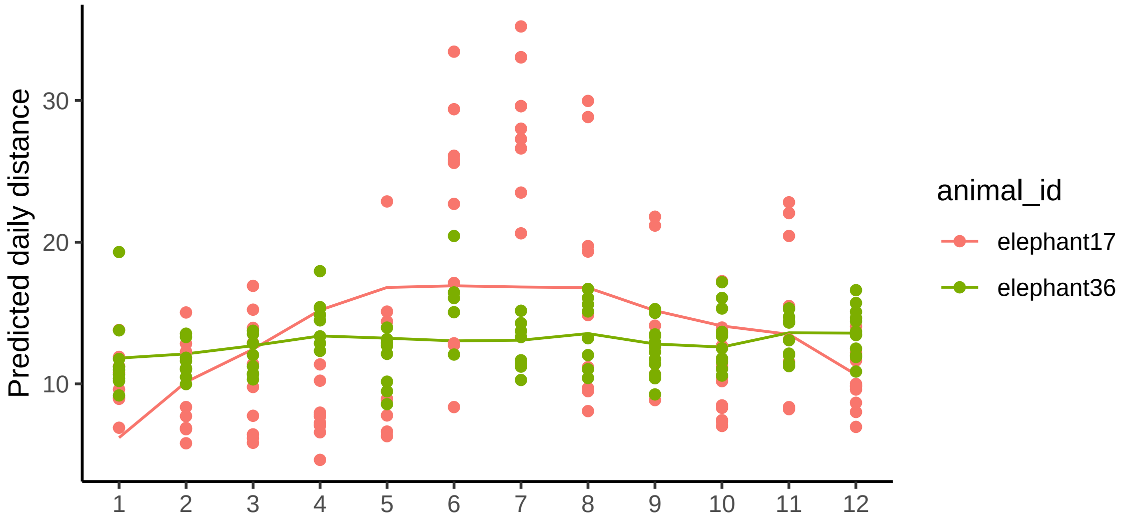

Figure 15: Observed data and model predictions for the most and least predictable elephant.

Correlation between intra-individual variability and behavioral types

As a simple extention of the above introduced DHGLM we can also estimate the correlation between residual intra-individual variance (i.e. an individual's variance around its trait mean) and behavioral types (i.e. an individual's trait mean). Such a correlation can give insights whether certain behavioral types are particularly predictable or un-predictable in their behavior. We can fit such a correlation by adding a term between the specified random slope and intercept terms in both the mean part oft the model, and the variance part. Below we chose to add the character "|a", the character can be randomly chosen.

```
double_model_cor = bf(meanDailyDisplacement ~
                      poly(month,2) + Sex + (poly(month,2)|a|animal_id),
                    sigma ~ (1|a|animal_id))

m3_brm_cor <- brm(double_model_cor,
              data    = data,
              warmup = 500,
              iter = 3000,
              thin=2,
              chains = 2,
              inits   = "random",
              seed = 12345,
              cores   = my.cores)
```

```
m3_brm_cor <- readRDS("m3_brm_cor.rds")
summary(m3_brm_cor)
```

```
##  Family: gaussian
##   Links: mu = identity; sigma = log
## Formula: meanDailyDisplacement ~ poly(month, 2) + Sex + (poly(month, 2) | a | animal_id)
##          sigma ~ (1 | a | animal_id)
##    Data: data (Number of observations: 3766)
## Samples: 2 chains, each with iter = 3000; warmup = 500; thin = 2;
##          total post-warmup samples = 2500
##
## Group-Level Effects:
## ~animal_id (Number of levels: 35)
##                                  Estimate Est.Error l-95% CI u-95% CI Rhat Bulk_ESS Tail_ESS
```

```
## sd(Intercept)                        2.42      0.32     1.89     3.15 1.01      819     1247
## sd(polymonth21)                     66.89      9.77    50.70    88.53 1.00     1147     1689
## sd(polymonth22)                     74.04     10.62    56.05    97.17 1.01     1324     1758
## sd(sigma_Intercept)                  0.27      0.04     0.21     0.35 1.01     1317     1547
## cor(Intercept,polymonth21)          -0.06      0.16    -0.37     0.26 1.00     1289     1513
## cor(Intercept,polymonth22)           0.24      0.16    -0.09     0.53 1.00     1071     1782
## cor(polymonth21,polymonth22)         0.01      0.17    -0.31     0.34 1.00     1090     1551
## cor(Intercept,sigma_Intercept)       0.48      0.15     0.17     0.73 1.00      920     1331
## cor(polymonth21,sigma_Intercept)    -0.16      0.17    -0.47     0.18 1.00     1240     1584
## cor(polymonth22,sigma_Intercept)    -0.26      0.16    -0.54     0.07 1.00     1132     1928
##
## Population-Level Effects:
##                 Estimate Est.Error l-95% CI u-95% CI Rhat Bulk_ESS Tail_ESS
## Intercept          14.21      0.54    13.19    15.32 1.00      540     1129
## sigma_Intercept     1.34      0.05     1.24     1.43 1.00      781     1165
## polymonth21       -16.52     12.02   -40.05     7.09 1.00      967     1480
## polymonth22        37.37     13.16    12.16    62.89 1.00      996     1433
## SexM               -2.95      0.75    -4.50    -1.49 1.01      582      851
##
## Samples were drawn using sampling(NUTS). For each parameter, Bulk_ESS
## and Tail_ESS are effective sample size measures, and Rhat is the potential
## scale reduction factor on split chains (at convergence, Rhat = 1).
```

We now find an additional term "cor(Intercept,sigma_Intercept)" under "Group-Level Effects". "cor(Intercept,sigma_Intercept)" is the term that estimates whether average behavior (behavioral type) and residual variance (predictability) are correlated. As we can see in the model output, there is a positive correlation between "Intercept" and "sigma_Intercept" which is estimated at 0.5 [0.18, 0.74]. Elephants with longer daily travel distances are therefore also less predictable than elephants with shorter movement distances.

Behavioral syndromes: Multivariate mixed effects models

Behavioral syndromes are among-individual correlations of two or more behaviors, i.e. the correlation between each individuals estimated average trait values. Behavioral syndromes were formerly analyzed using classical multivariate modelling techniques, like PCA or NMDS, on BLUPs extracted from univariate models (see section 1) of the respective two or more behaviors. More recently these approaches have come under criticism (see e.g. T.M. Houslay, A.J. Wilson (2017). Avoiding the misuse of BLUP in behavioral ecology. Behavioral Ecology, 28(4), 948-952. doi: 10.1093/beheco/arx023 for an extended discussion). The state of the art approach for analyzing behavioral syndromes are multivariate mixed models which can cope with multiple response variables at the same time and estimate their correlation on the random effect (e.g. individual) level. Mixed effects models are also very robust for differences in sample sizes (i.e. number of measurements) between each individual. The R packages MCMCglmm and brms are regularly used to fit such models.

We here will use the brms package for model fitting. You can find more information on prior and model specification in MCMCglmm in under https://tomhouslay.com/tutorials/ and in Hadfield (2010) "MCMC methods for multi-response generalized linear mixed models: the MCMCglmm R package."

For the simplicity of this tutorial we will drop the random effects of "year" and "month". The three behaviors are on very different scales. Travel distance in km, residence time in hours and turn angle correlation are on very different scales. For better model fitting we will *scale* behaviors, meaning that we center each behavior at its mean value and standardize it to units of 1 phenotypic standard deviation. As all three behaviors are now on the same scale, it is easier to interpret the model estimates.

```
m4_brm <- brm(mvbind(scale(meanDailyDisplacement),scale(tac),scale(meanRT)) ~
              Sex + poly(month,2) + (1|p|animal_id),
              data = data, family = "gaussian",
              warmup = 500,iter = 3000, thin=2,
```

```
              chains = 2, inits  = "random",
              seed = 12345,
              cores  = my.cores)
```

We can access posterior means and credible intervals using the summary() call.

```
m4_brm <- readRDS("m4_brm.rds")
summary(m4_brm)
```

```
##  Family: MV(gaussian, gaussian, gaussian)
##   Links: mu = identity; sigma = identity
##          mu = identity; sigma = identity
##          mu = identity; sigma = identity
## Formula: scale(meanDailyDisplacement) ~ Sex + poly(month, 2) + (1 | p | animal_id)
##          scale(tac) ~ Sex + poly(month, 2) + (1 | p | animal_id)
##          scale(meanRT) ~ Sex + poly(month, 2) + (1 | p | animal_id)
##    Data: data (Number of observations: 3759)
## Samples: 2 chains, each with iter = 3000; warmup = 500; thin = 2;
##          total post-warmup samples = 2500
##
## Group-Level Effects:
## ~animal_id (Number of levels: 35)
##                                                                 Estimate Est.Error l-95% CI u-95% CI Rhat Bulk_ESS Tail_ESS
## sd(scalemeanDailyDisplacement_Intercept)                            0.48      0.06     0.37     0.61 1.00     1221     1806
## sd(scaletac_Intercept)                                              0.51      0.07     0.39     0.66 1.00     1070     1714
## sd(scalemeanRT_Intercept)                                           0.41      0.06     0.31     0.53 1.00     1465     1931
## cor(scalemeanDailyDisplacement_Intercept,scaletac_Intercept)       -0.03      0.18    -0.37     0.30 1.00     1053     1449
## cor(scalemeanDailyDisplacement_Intercept,scalemeanRT_Intercept)    -0.42      0.15    -0.68    -0.08 1.00     1418     1843
## cor(scaletac_Intercept,scalemeanRT_Intercept)                       0.22      0.17    -0.11     0.53 1.00     1393     1458
##
## Population-Level Effects:
##                                        Estimate Est.Error l-95% CI u-95% CI Rhat Bulk_ESS Tail_ESS
## scalemeanDailyDisplacement_Intercept       0.18      0.11    -0.04     0.40 1.00     1022     1286
## scaletac_Intercept                         0.37      0.12     0.15     0.60 1.00     1086     1383
## scalemeanRT_Intercept                      0.13      0.09    -0.06     0.33 1.00     1474     1573
## scalemeanDailyDisplacement_SexM           -0.46      0.16    -0.79    -0.14 1.00     1158     1320
## scalemeanDailyDisplacement_polymonth21    -1.99      0.87    -3.66    -0.31 1.00     2523     2419
## scalemeanDailyDisplacement_polymonth22     8.11      0.88     6.38     9.81 1.00     2475     2313
## scaletac_SexM                             -0.78      0.18    -1.14    -0.43 1.00      941     1280
## scaletac_polymonth21                      10.02      0.73     8.63    11.45 1.00     2332     2148
## scaletac_polymonth22                     -16.67      0.76   -18.18   -15.20 1.00     2326     2209
## scalemeanRT_SexM                          -0.15      0.14    -0.43     0.12 1.00     1239     1702
## scalemeanRT_polymonth21                    3.99      0.92     2.18     5.91 1.00     2380     2284
## scalemeanRT_polymonth22                    3.96      0.92     2.18     5.70 1.00     2391     2175
##
## Family Specific Parameters:
##                                  Estimate Est.Error l-95% CI u-95% CI Rhat Bulk_ESS Tail_ESS
## sigma_scalemeanDailyDisplacement     0.86      0.01     0.84     0.88 1.00     2332     2108
## sigma_scaletac                       0.75      0.01     0.74     0.77 1.00     2383     2236
## sigma_scalemeanRT                    0.92      0.01     0.90     0.94 1.00     2198     1740
##
## Residual Correlations:
##                                               Estimate Est.Error l-95% CI u-95% CI Rhat Bulk_ESS Tail_ESS
## rescor(scalemeanDailyDisplacement,scaletac)       0.26      0.02     0.23     0.29 1.00     2381     2392
## rescor(scalemeanDailyDisplacement,scalemeanRT)    0.03      0.02    -0.00     0.06 1.00     2097     1882
## rescor(scaletac,scalemeanRT)                     -0.12      0.02    -0.15    -0.09 1.00     2293     1989
##
## Samples were drawn using sampling(NUTS). For each parameter, Bulk_ESS
## and Tail_ESS are effective sample size measures, and Rhat is the potential
## scale reduction factor on split chains (at convergence, Rhat = 1).
```

The model output contains information how each behavior is affected by the fixed effects, independently from the other two behaviors ("Population-level Effects"). The output also contains information on the variance explained by the random effect of individual in each behavior ("Group-Level Effects, terms with"sd" in the beginning) and about the correlation between behaviors (terms with "cor" in the beginning). Finally the section "Family Specific Parameters" contains the residual variance for every behavior.

Lets try to make sense how we can calculate the repeatability of of each behavior (similar to a univariate model).Lets recalculate repeatability for movement distance. Mind, we are using indexing here because column names are becoming very long. For example, when selecting the column containing the posterior distribution of the variance of animal_id: "sd__animal__id___scalemeanDailyDisplacement_Intercept" we simply use the index of the column [,13]. Mind again, different from MCMCglmm, in brms variance parameters are in standard deviations and need to be squared!

```
colnames(posterior_samples(m4_brm))[1:18]
```

```
##  [1] "b_scalemeanDailyDisplacement_Intercept"
##  [2] "b_scaletac_Intercept"
##  [3] "b_scalemeanRT_Intercept"
##  [4] "b_scalemeanDailyDisplacement_SexM"
##  [5] "b_scalemeanDailyDisplacement_polymonth21"
##  [6] "b_scalemeanDailyDisplacement_polymonth22"
##  [7] "b_scaletac_SexM"
```

```
##  [8] "b_scaletac_polymonth21"
##  [9] "b_scaletac_polymonth22"
## [10] "b_scalemeanRT_SexM"
## [11] "b_scalemeanRT_polymonth21"
## [12] "b_scalemeanRT_polymonth22"
## [13] "sd_animal_id__scalemeanDailyDisplacement_Intercept"
## [14] "sd_animal_id__scaletac_Intercept"
## [15] "sd_animal_id__scalemeanRT_Intercept"
## [16] "cor_animal_id__scalemeanDailyDisplacement_Intercept__scaletac_Intercept"
## [17] "cor_animal_id__scalemeanDailyDisplacement_Intercept__scalemeanRT_Intercept"
## [18] "cor_animal_id__scaletac_Intercept__scalemeanRT_Intercept"
```

```
var.animal.dist <- posterior_samples(m4_brm)[,13]^2
var.res.dist <- posterior_samples(m4_brm)[,19]^2

var.tot.dist <- var.animal.dist + var.res.dist

RDist <- var.animal.dist / var.tot.dist
mean(RDist);HPDinterval(as.mcmc(RDist),0.95)
```

```
## [1] 0.2346999
```

```
##          lower     upper
## var1 0.1456801 0.3241302
## attr(,"Probability")
## [1] 0.95
```

As expected, the repeatability of behavior (here daily movement distance) is equal to the repeatability calculated from a univariate model (section 1).

The brms package already calculates the mean and credible interval of the among-individual correlation between behaviors for us and prints it in the model summary. We can access and plot the posterior distribution of this correlation. If the credible interval (dashed lines in Fig 15) does not include 0 (indicated in red) we can assume a significant correlation.

```
Dist_RT <- posterior_samples(m4_brm)[,17]

par(mar=c(2, 2, 3, 2))
plot(density(Dist_RT),
     main="Correlation Distance - RT",
     xlab="",ylab="",cex.main=0.8)
abline(v=0,col="red",lwd=2)
abline(v=mean(Dist_RT),col="black",lwd=2)
abline(v=HPDinterval(as.mcmc(Dist_RT))[1],col="black",lty=2)
abline(v=HPDinterval(as.mcmc(Dist_RT))[2],col="black",lty=2)
```

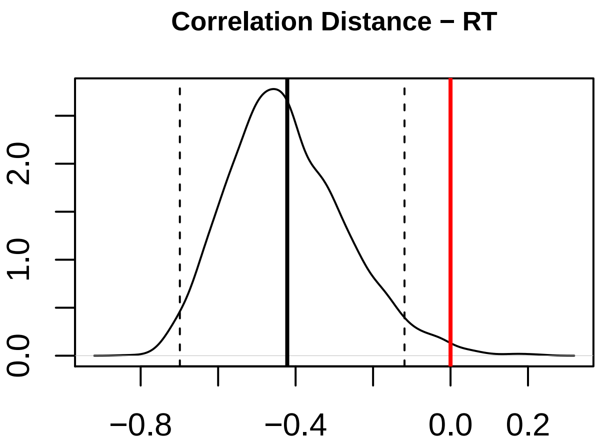

Figure 16: Correlation between mean behavioral expression of movement distance and residence time. Dashed lines indicate the 95% credible interval of the posterior distribution.

Daily movement distance and residence time are negatively correlated (cor = -0.4) on the individual level meaning that individuals that travel farther also have shorter average residence times at a location. Examining the posterior distribution, the credible intervals are really wide, most likely because multivariate mixed models are very data hungry.
In the summary output we already see that the correlation between daily movement distance and turn angle correlation (mean = -0.02, credible interval = [-0.36, 0.32]), and between residence time and turn angle (mean = 0.22, credible interval = [-0.13, 0.52]) both overlap with 0, indicating that there is no functional correlation between the respective two movement traits on the individual level. For demonstrative purposes we can anyways go ahead and plot the among individual correlation of behaviors. For this we need to extract the posterior mean behavioral types (BLUP) and calculate the slope between behaviors.

```
BLUP <- data_frame(Trait = names(posterior_samples(m4_brm)[,25:129]),
                   Value = colMeans(posterior_samples(m4_brm)[,25:129])) %>%
  separate(Trait, c(NA,NA,NA,NA,"Trait","animal_id",NA),
           sep = "([\\__\\[\\,])", fill = "right") %>%
  spread(Trait, Value)

head(BLUP)
```

```
## # A tibble: 6 x 4
##   animal_id  scalemeanDailyDisplacement scalemeanRT scaletac
##   <chr>                           <dbl>       <dbl>    <dbl>
## 1 elephant1                       0.526    0.000440   0.290
## 2 elephant10                      0.489   -0.501      0.0543
## 3 elephant11                     -0.317   -0.548     -0.632
## 4 elephant13                      1.02    -0.0978    -0.0697
## 5 elephant15                     -0.470    0.0960     0.160
## 6 elephant16                      0.222   -0.231      0.0798
```

For the slope we divide the covariance between the two focal behaviors by the variance of the one to be plotted on the x - axis, where the covariance is calculated as

$$COV_{(X,Y)} = (COR_{X,Y} * sqrt(Var_X) * sqrt(Var_Y)$$

For example, the slope between movement distance and residence time would be calculated as follows:

```
cov.DIST_RT <-
  posterior_samples(m4_brm)[,17] *
```

```
  sqrt(posterior_samples(m4_brm)[,15]^2) *
  sqrt(posterior_samples(m4_brm)[,13]^2)

var.Dist <- posterior_samples(m4_brm)[,13]^2

DIST_RT_slope <- cov.DIST_RT / var.Dist
```

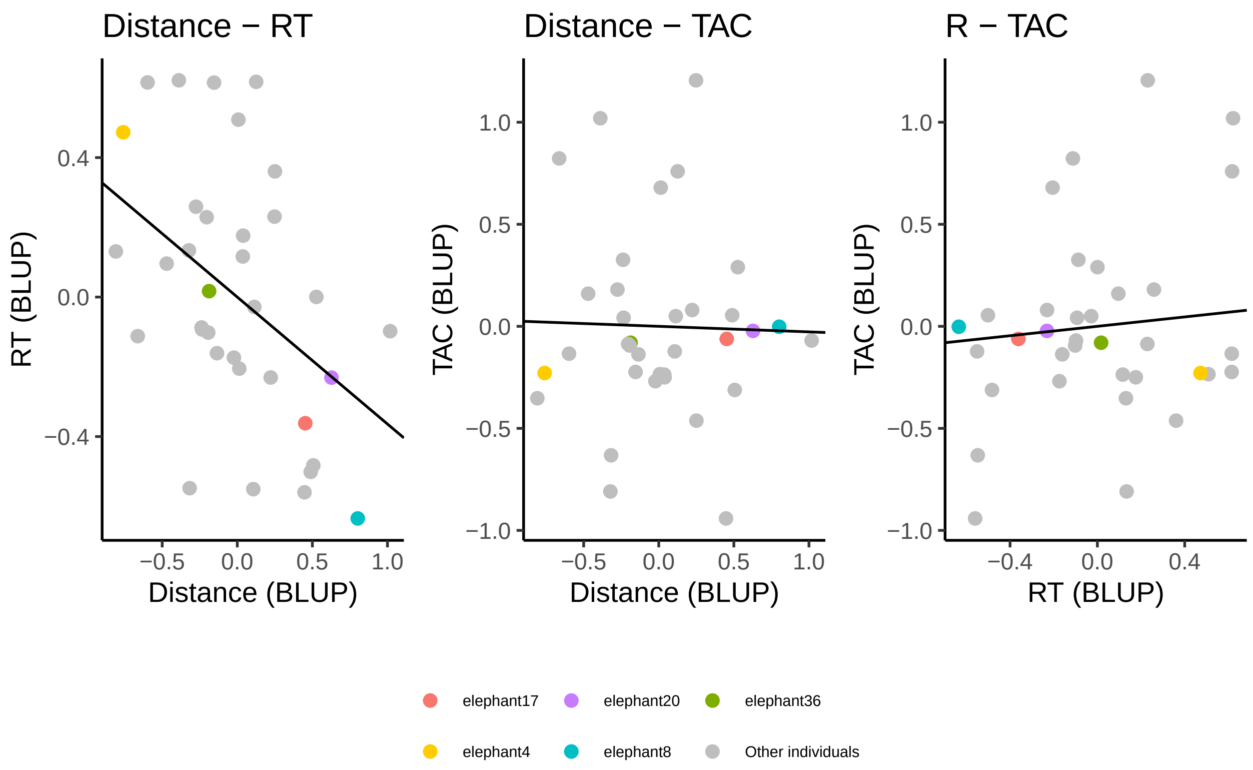

Figure 17: Behavioral syndrome between Daily movement distance, residence time (RT), and turn angle correlation (TAC)

In Fig 16 we clearly see the negative correlation between daily movement distance and residence time and a lack of such correlation between the other two combinations of behavior. Mind, because we scaled behaviors in the beginning it is however not as straight forward as in section 1 to add estimates of population level effects (Intercept and Sex, resp.) to BLUP's to calculate the actual behavior (e.g. movement distance in km).

Concluding remarks

Using tracking data of 35 African elephants from Etosha Nationalpark in Namibia we could show that, after controlling for two basic fixed effects - month of the year and sex of the elephant - individuals differed consistently in three simple movement traits quantified on a weekly basis: weekly means of daily movement distance, mean residence time at any given location within a week, and turn angle correlation over all successive steps within a week. We did not control for a range of other parameters that are likely to affect behavior, e.g. habitat composition, presence of other elephants and herd size (which is variable over time), age of the elephant etc. In a systematic study other *fixed effects* that are known or expected to affect behavior need to be controlled for. The intention of this example is to be a "how to" guide for ecologists. For a research example that attempts to draw actual conclusions on individual variation in elephant behavior one would need to include more covariates.

We further found that elephants differed in how they adjusted behavior over seasonal changes. Elephants that moved over shorter daily distances during the dry season than their conspecifics had the tendency to either further decrease their movement during the wet season or to increase it less strongly than their more mobile conspecifics which increased movement during the wet as compared to the dry season. We further found evidence that indeed, elephants differed how they changed their behavior over the entire year, making a strong case for random regression over month of the year. After controlling for individual differences in intercept and plasticity over the course of the year, elephants still differed in how predictable they were in their behavior, calculated as individual specific residual variance. While some elephants had a high residual variance and were unpredictable in their behavior over the time evaluated, others were much more predictable. Out of the three movement traits evaluated, two were negatively correlated on the individual level - elephants with shorter daily movement distance also had longer residence times. Turn angle correlation was uncorrelated to movement distance or residence time.

We hope that this tutorial may stimulate wildlife ecologists to explore their data for consistency in individual variation of movement traits to answer novel and biologically more detailed questions.

Author affiliation and Funding

Please direct any questions or comments to anne.hertel@senckenberg.de

At the time of publication Anne G. Hertel is affiliated with the Senckenberg Biodiversity and Climate Research Centre (SBiK-F) in Frankfurt (Main), Germany, and the Department of Environmental and Health Studies, University College of Southeast Norway in Bø, Norway.

AGH has received funding from the European Union's Horizon 2020 research and innovation programme under the Marie Skłodowska-Curie grant agreement No 793077.

Acknowledgements

We thank Wayne Getz, Werner Kilian, and Briana Abrahms for providing the movement dataset on African Elephants in Etosha Nationalpark.
